# Small hypoxia-primed mesenchymal stem cells attenuate graft-versus-host disease

**DOI:** 10.1038/s41375-018-0151-8

**Published:** 2018-05-22

**Authors:** YongHwan Kim, Hye Jin Jin, Jinbeom Heo, Hyein Ju, Hye-Yeon Lee, Sujin Kim, Seungun Lee, Jisun Lim, Sang Young Jeong, JiHye Kwon, Miyeon Kim, Soo Jin Choi, Wonil Oh, Yoon Sun Yang, Hyun Ho Hwang, Hwan Yeul Yu, Chae-Min Ryu, Hong Bae Jeon, Dong-Myung Shin

**Affiliations:** 1 Department of Biomedical Sciences, Asan Medical Center, University of Ulsan College of Medicine, Seoul, 05505 Korea; 2 Department of Physiology, University of Ulsan College of Medicine, Seoul, 05505 Korea; 3 Biomedical Research Institute, MEDIPOST Co., Ltd, Seongnam, 13494 Korea; 4 King Abdullah University of Science and Technology (KAUST), Thuwal, Jeddah 23955-6900 Saudi Arabia

## Abstract

Mesenchymal stem cells (MSCs) are of particular interest for the treatment of immune-related diseases due to their immunosuppressive capacity. Here, we show that Small MSCs primed with Hypoxia and Calcium ions (SHC-MSCs) exhibit enhanced stemness and immunomodulatory functions for treating allogeneic conflicts. Compared with naïve cultured human umbilical cord blood-derived MSCs, SHC-MSCs were resistant to passage-dependent senescence mediated via the monocyte chemoattractant protein-1 and p53/p21 cascade and secreted large amounts of pro-angiogenic and immunomodulatory factors, resulting in suppression of T-cell proliferation. SHC-MSCs showed DNA demethylation in pluripotency, germline, and imprinted genes similarly to very small embryonic-like stem cells, suggesting a potential mutual relationship. Genome-wide DNA methylome and transcriptome analyses indicated that genes related to immune modulation, cell adhesion, and the cell cycle were up-regulated in SHC-MSCs. Particularly, polo-like kinase-1 (*PLK1*), zinc-finger protein-143, dehydrogenase/reductase-3, and friend-of-GATA2 play a key role in the beneficial effects of SHC-MSCs. Administration of SHC-MSCs or *PLK1*-overexpressing MSCs significantly ameliorated symptoms of graft-versus-host disease (GVHD) in a humanized mouse model, resulting in significantly improved survival, less weight loss, and reduced histopathologic injuries in GVHD target organs compared with naïve MSC-infused mice. Collectively, our findings suggest that SHC-MSCs can improve the clinical treatment of allogeneic conflicts, including GVHD.

## Introduction

Although advances in allogeneic hematopoietic stem cell (SC) transplantation have improved the overall survival of patients with certain malignant and nonmalignant diseases, graft-versus-host disease (GVHD) remains a leading cause of late morbidity and mortality even following transplantation of cells acquired from human leukocyte antigen (HLA)-matched siblings [1, 2]. Most cases of GVHD are caused by the reaction of donor T-cells with histo-incompatible antigens of the recipient. The ensuing proliferation or activation of other immune cells leads to a wide variety of host tissue injuries caused by the release of inflammatory cytokines [3]. The standard first-line therapy for GVHD is a high dose of steroids with or without a calcineurin inhibitor to shorten the duration of steroid use [3]. However, about 50% of patients do not respond to first-line treatment and the efficacy of current treatment options is severely limited in patients with steroid-refractory disease [3]. Unfortunately, there are no standards of care or agents approved by the United States Food and Drug Administration/European Medicines Agency for the second-line treatment of steroid-refractory GVHD, and novel therapies need to be urgently developed [3].

Therapy using mesenchymal SCs (MSCs) derived from umbilical cord blood (UCB) or various adult tissues including bone marrow (BM) is a promising strategy for treating incurable GVHD [4–8] based on the immunomodulatory functions of these cells [9]. In particular, MSCs inhibit the activation, proliferation, and function of immune cells, including T-cells, B-cells, natural killer cells, and antigen-presenting cells [10]. MSCs induce immunosuppression via cell contact-dependent mechanisms involving B7-H1 and soluble factors such as interleukin (IL)-10, transforming growth factor-β, nitric oxide, prostaglandin E2 (PGE2), and indoleamine 2,3-dioxygenase [11, 12]. Furthermore, due to their multipotency, MSCs can be used to replace host cells in the microenvironment of target tissues that have been damaged by chemotherapy or irradiation. MSCs can also provide growth factors, mediate cell-cell interactions, and supply matrix proteins to modulate the microenvironment of damaged target tissues and thereby facilitate regeneration [13].

Several preclinical and clinical studies have reported beneficial effects of MSCs on GVHD [4, 5]. However, the clinical application of these cells is hindered by their limited therapeutic efficacy and technical problems associated with large-scale ex vivo expansion to obtain a sufficient number of cells with a similar therapeutic potential. Indeed, primitive MSCs with a high therapeutic potency, which are maintained in vivo by residing in specific niches, are extremely unstable in vitro due to the frequent accumulation of epigenetic abnormalities and oxidative stress provoked by supra-physiological stimulations [14, 15]. Therefore, determination of the optimal culture conditions for primitive MSCs in vitro and identification of related morphologic and molecular parameters will not only help to develop strategies for the optimal and safe use of MSCs for therapeutic purposes, but also improve understanding of the developmental hierarchy of SCs [16].

Evidence has accumulated that cell size greatly affects cell transplantation-based regenerative therapy [16]. Among MSC subpopulations with differing self-renewal capacities and therapeutic properties, small SCs readily self-renew, whereas large flattened cells tend to lose SC characteristics [17]. MSCs cultured as three-dimensional (3D) spheroids are up to 75% smaller than their two-dimensional counterparts, and these small cells elicit enhanced anti-inflammatory, pro-angiogenic, and tissue-regenerative effects and survive better after transplantation [18]. Tissue capillaries have a diameter of around 8 μm and trap more than 85% of systematically injected MSCs. Small SCs are less likely to become trapped in capillaries after transplantation, which would be beneficial for clinical applications.

Furthermore, very small embryonic-like SCs (VSELs), whose diameter is <6 μm, are the most primitive population of quiescent SCs in adult tissues and can differentiate into cells of all three germ layers [19]. VSELs are very rare (~0.01% of nucleated BM cells) and have unique gene expression and epigenetic profiles, including an open/active chromatin structure in the *OCT4* promoter and DNA demethylation in paternally imprinted genes, which maintain pluripotency and quiescence, respectively [20]. Small primitive SCs including VSELs, which have been observed by several independent investigators, may be precursors of a wide range of adult SCs [16].

In this regard, the isolation and cultivation of small SCs that retain primitive characteristics may overcome the drawbacks of current MSC-based therapies. We recently reported that exposure to mild hypoxia (∼5% O_2_) during the isolation and ex vivo expansion of human UCB-derived MSCs (UCB-MSCs) enriches highly primitive SCs and improves the therapeutic efficacy for ameliorating asthmatic inflammatory injuries [21]. In addition, treatment with an elevated concentration of calcium ions (Ca^2+^) enhances the proliferation and differentiation capacities of UCB-MSCs [22]. In this study, we improved a wide range of MSC functions, including their proliferative, self-renewal, migratory, pro-angiogenic, anti-inflammatory, and immunomodulatory capacities, using a one-step process termed Small cells primed with Hypoxia and Calcium ions (SHC) that does not involve genetic manipulation. Transcriptome and DNA methylome analyses revealed that the genes responsible for these effects included polo-like kinase-1 (*PLK1*), zinc-finger protein-143 (*ZNF143*), friend-of-GATA2 (*FOG2*), and dehydrogenase/reductase-3 (*DHRS3*). Finally, we demonstrated the in vivo significance of this procedure by showing that SHC-MSCs and *PLK1*-overexpressing MSCs exhibited an enhanced therapeutic potency in a humanized mouse model of GVHD.

## Methods

### Cell culture

**Fig. 1:**
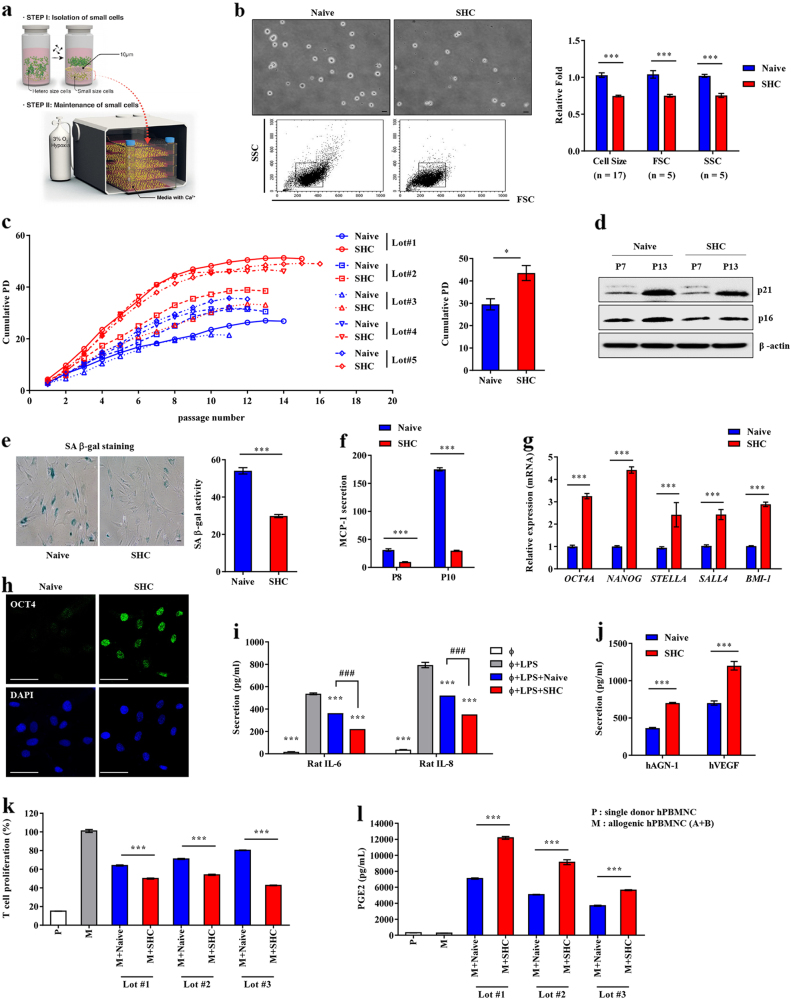
Enhanced anti-inflammatory and immunomodulatory functions of small MSCs enriched via the SHC procedure (**a**) Schematic summary of the SHC procedure. **b** Cell size was measured by microscopy and flow cytometry (×200 magnification, scale bar = 10 μm). The flow cytometric results were quantified, and the size of SHC-MSCs is shown relative to that of naïve MSCs (set to 1-fold, *n* ≥ 5). **c** Growth kinetics (left panel) and cumulative PD (right panel, *n* = 5) of naïve and SHC-MSCs from five independent donors. PD was monitored until cells stopped proliferating. **e** Representative images (left panel, ×100 magnification, scale bar = 50 μm) and quantification (right panel, *n* = 10) of SA β-gal staining of naïve and SHC-MSCs at P10. **d**, **f** Expression of senescence-related proteins (**d**) and secretion of MCP-1 (**f**, *n* = 3) in naïve MSCs and SHC-MSCs at intermediate (P7 or P8) and late (P10 or P13) passage numbers. **g**, **h** Quantification of mRNA expression of primitive SC genes (*OCT4A*, *NANOG*, *STELLA, SALL4*, and *BMI-1*) (**g**, *n* = 6) and immunofluorescence staining of OCT4 protein (**h**, green, ×400 magnification, scale bar = 50 μm) in naïve and SHC-MSCs at P5. Nuclei were counterstained with Hoechst 33342 (blue). **i**, **j** Levels of rat pro-inflammatory cytokines (IL-6 and IL-8) (**i**, *n* = 3) and human anti-inflammatory proteins (ANG-1 and VEGF) (**j**, *n* = 3) in CM of LPS-stimulated rat alveolar macrophages (ϕ + LPS) co-cultured with naïve MSCs (ϕ + LPS + Naïve) or SHC-MSCs (ϕ + LPS + SHC). **k** Proliferation of human T-cells in the MLR assay (*n* = 3). PBMNCs were co-cultured with naïve MSCs or SHC-MSCs. The proliferation of responding cells is shown as a percentage relative to the positive control (M; set to 100%). **l** Level of PGE2 in the CM of cells in the MLR assay (*n* = 3). Three independent lots of UCB-MSCs were used in the experiments. Data are mean ± SEM. *p < 0.05, ***p < 0.001, ^###^*p* < 0.001, Mann–Whitney *U* test, one-way or two-way ANOVA with the Bonferroni post-test

This study was approved by the Institutional Review Board of MEDIPOST Co., Ltd. (P01-201601-31-003). Collection of human UCB and isolation and culture of UCB-MSCs were performed as previously described [21, 23]. Mononuclear cells were isolated by centrifugation through a Ficoll-Hypaque gradient (density, 1077 g/cm^2^ ; Sigma-Aldrich, St. Louis, MO), washed, seeded at a density of 5 × 10^5^ cells/cm^2^, and maintained in hypoxic conditions (3% O_2_) in Minimum Essential Medium-α (MEM-α; Gibco, Carlsbad, Grand Island, NY) supplemented with 1.8 mM calcium and 10% fetal bovine serum (FBS; Gibco). MSC colonies were trypsinized and counted after fibroblast-like adherent cells formed. For the SHC procedure (Fig. 1a), MSCs at a density of 1 × 10^5^ cells/mL were filtered through a pluriStrainer with a pore size of 10 μm (pluriSelect, San Diego, CA) to enrich small cells with a minimal risk of cell damage or contamination. Small MSCs were counted, reseeded at a density of 800–2000 cells/cm^2^, maintained under hypoxic conditions (3% O_2_) in MEM-α supplemented with 1.8 mM calcium and 10% FBS, and routinely cultured for 5 days. Cumulative population doubling (PD) was calculated for each passage based on the total number of cells [21]. This procedure was repeated until cells stopped proliferating. Five lots of UCB-MSCs were used. Basic information about UCB-MSCs is provided in Supplementary Table 1.

### Genome-wide gene expression and DNA methylation analyses

The detailed procedures used to analyze transcriptome and DNA methylome microarray data are described in Supplementary Methods. Functional analyses of the transcriptome and DNA methylome databases for gene networks, biofunctions, and canonical pathways were performed using MetaCore microarray software (Clarivate Analytics, Philadelphia, PA) or gene set enrichment analysis (GSEA; Broad Institute, Cambridge, MA) with default settings. The data described in this study have been deposited in the Gene Expression Omnibus of the NCBI and are accessible under GEO Series accession number GSE108564.

### A humanized GVHD animal model

All animal experiments were approved by the Institutional Animal Care and Use Committee of the University of Ulsan College of Medicine (IACUC-2016-12-325). The animal model of GVHD was generated as described previously [8]. In brief, 9-week-old male non-obese diabetic (NOD). Cg-*Prkdc*^scid^ *Il2rg*^*tm1Wjl*^/SzJ (NSG) mice (26–29 g) were irradiated (2.0 Gy) and injected with 2.5 × 10^6^ human peripheral blood mononuclear cells (PBMNCs) or the same volume of phosphate-buffered saline (PBS; Sham group) via the tail vein within 24 h after irradiation. After 18 days, 5 × 10^5^ naïve MSCs, SHC-MSCs, or *PLK1*-overexpressing MSCs suspended in 100 μL of PBS were injected via the tail vein. PBS alone (Sham and GVHD groups) was injected as a control. Clinical symptoms of GVHD were evaluated daily by examining body weight loss, survival, hunched back, and fur texture and recorded every second day. Five mice per group were used in two independent experiments. Six weeks after MSC administration, target organs (lung, liver, kidney, and small intestine) and blood plasma were harvested from mice in all groups for histological and cytokine analyses as described in Supplementary Methods. Mice were randomly allocated to the groups. In each group, mice were irradiated, transplanted with cells or injected with vehicle, and examined daily in random orders. The type of injected MSCs was masked from investigators involved in the GVHD induction procedures. Clinical symptoms, histological staining, and gene expression were assessed by investigators who were blinded to the treatment groups.

### Statistical analysis

Data were statistically analyzed using the non-parametric Mann–Whitney test or a one-way ANOVA with the Bonferroni post-hoc test. All analyses were performed using GraphPad Prism 6.0 software (GraphPad Software, La Jolla, CA). *p* < 0.05 was considered statistically significant.

Details of other experimental procedures are described in Supplementary Methods.

## Results

### The SHC procedure enriches primitive MSCs

We developed a simple protocol (Fig. 1a), termed SHC, to enrich small MSCs and stimulate their functions via exposure to hypoxia and an elevated level of Ca^2+^. Fluorescence-activated cell sorting analysis confirmed that the majority of SHC-MSCs (92.3 ± 5.8%) had a diameter of ≤10 μm and that SHC-MSCs were ~0.6-fold smaller than naïve MSCs (Fig. 1b and Supplementary Fig. 1a). Expression of MSC surface marker proteins (CD73, CD90, CD105, and CD166) and multipotency, as assessing by alkaline phosphatase (osteogenesis), Safranin O (chondrogenesis), and Oil Red O (adipogenesis) staining, did not markedly differ between SHC-MSCs and naïve MSCs (Supplementary Fig. 1b,c). As expected, naïve MSCs and SHC-MSCs barely expressed hematopoietic lineage markers (CD14 and CD45) and the MHC class II protein HLA-DR.

Upon long-term expansion over 16 passages, SHC-MSCs from five donors were remarkably resistant to replicative senescence, and thus the cumulative PD of SHC-MSCs was higher than that of naïve MSCs (Fig. 1c). Consistently, SHC-MSCs did not exhibit an enlarged morphology or strong senescence-associated β-galactosidase (SA-β-gal) staining (Fig. 1e) and did not highly express senescence-associated proteins such as the cyclin-dependent kinase inhibitors p21^CIP1^ and p16^INK4a^ (Fig. 1d). We previously reported that senescence of UCB-MSCs is orchestrated by the chemokine monocyte chemoattractant protein-1 (MCP-1; also known as C-C motif ligand-2), which is secreted as a major component of the senescence-associated secretory phenotype and is epigenetically regulated by BMI-1 [21]. The increase in MCP-1 secretion upon repeated passage was remarkably repressed in SHC-MSCs (Fig. 1f). SHC-MSCs increased clonogenic activity in the colony-forming unit-fibroblast (CFU-F) assay (Supplementary Fig. 2a), expressed the surface markers CXCR4, CD49f, and CD146, which are characteristic of primitive MSCs [24, 25] (Supplementary Fig. 2b,c), and expressed genes related to primitive SCs including *OCT4*, *NANOG*, *STELLA*, *SALL4*, and *BMI-1* (Fig. 1g). We confirmed that SHC-MSCs expressed *OCT4A*, a pluripotency-specific transcript, by DNA sequencing (Supplementary Fig. 2d). Moreover, immunofluorescence staining demonstrated the expression of OCT4 protein localized to the nuclei of SHC-MSCs (Fig. 1h). In addition, the *OCT4* promoter was enriched with histone modifications indicative of an open chromatin structure in SHC-MSCs in comparison with naïve MSCs (Supplementary Fig. 2e).

We next compared the anti-inflammatory and immunomodulatory properties of naïve MSCs and SHC-MSCs. For the in vitro anti-inflammatory assay, rat alveolar NR8383 macrophages stimulated with lipopolysaccharide (LPS) were co-cultured with naïve MSCs or SHC-MSCs derived from three donors. Secretion of the pro-inflammatory cytokines IL-6 and IL-8 was increased in LPS-stimulated NR8383 cells; however, this was significantly inhibited by co-culture with naïve MSCs or SHC-MSCs. The anti-inflammatory effect of SHC-MSCs was significantly superior to that of naïve MSCs (Fig. 1i). SHC-MSCs co-cultured with LPS-stimulated NR8383 macrophages secreted significantly higher levels of human angiopoitin-1 (ANG-1) and vascular epidermal growth factor (VEGF) (Fig. 1j), which are the main paracrine factors that protect against lung inflammation [26, 27], than naïve MSCs. In an allogenic mixed lymphocyte reaction (MLR) assay, MSCs from all three donors inhibited proliferation of PBMNCs in response to allogeneic stimulation, and the effect of SHC-MSCs was superior to that of naïve MSCs (Fig. 1k). To elucidate the contribution of soluble factors to this immunosuppressive effect, the supernatants of activated T-cells cultured in the absence or presence of MSCs were examined. Co-culture with SHC-MSCs increased secretion of PGE2 (Fig. 1l), a well-known soluble factor responsible for the immunoregulatory effects of MSCs [28]. Collectively, these results indicate that the SHC procedure can enrich small primitive SCs that are resistant to senescence and have improved self-renewal, anti-inflammatory, and immunomodulatory capacities.

### Genes related to immunomodulation, cell adhesion, and the cell cycle are up-regulated in SHC-MSCs

**Fig. 2:**
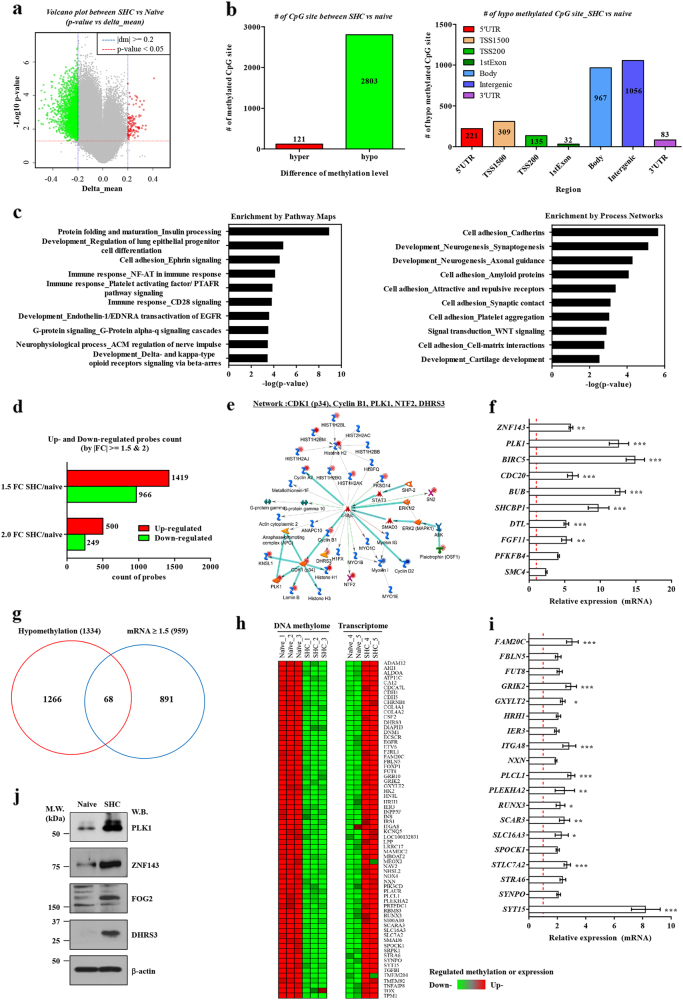
DNA methylome and transcriptome analysis of SHC-MSCs (**a**, **b**) Volcano plot (**a**) and numbers (**b**) of hyper-methylated and hypo-methylated CpG sites in SHC-MSCs versus naïve MSCs. The numbers of hypo-methylated CpG sites in each DNA element are indicated in the right panel. UTR, untranslated region; TSS, transcription start site. **c** The 10 most highly enriched Pathway Maps and Process Networks in MetaCore analysis. **d**, **e** Number of genes differentially expressed between naïve MSCs and SHC-MSCs (**d**) and a representative *PLK1* and *DHRS3-*associated gene network (**e**) identified via MetaCore analysis. Gene networks are illustrated by overlaying experimental values as fold changes in SHC-MSCs versus naïve MSCs. Up- and down-regulated genes are indicated in red and blue, respectively. **f** Real-time qPCR analysis of genes in the *PLK1-*associated, *DHRS3-*associated, *ZNF143*-associated networks. **g**, **h** Venn diagram (**g**) and heat-map (**h**) of 68 genes that were hypo-methylated and whose expression was increased (≥1.5-fold) in SHC-MSCs compared with naïve MSCs. **i**, **j** Real-time qPCR (**i**) and western blot (**j**) analyses of a subset of these 68 genes. In western blots, molecular weight (M.W.) marker sizes are shown on the left. β-actin was used as a loading control. Quantitative data show the fold change of expression in SHC-MSCs compared with that in naïve MSCs (set to 1; denoted by the red dotted line) (*n* = 4). *p < 0.05, **p < 0.01, ***p < 0.001, one-way ANOVA with the Bonferroni post-hoc test

To elucidate the molecular mechanisms underlying the effects of the SHC procedure, we compared the DNA methylomes of SHC-MSCs and naïve MSCs. At the genome-wide level, DNA was characteristically hypo-methylated in SHC-MSCs and the majority of hypo-methylated regions were located in the gene body and intergenic elements (Fig. 2a,b). Next, genes annotated as hypo-methylated sites were listed and their molecular characteristics were analyzed by the MetaCore pathway method. Gene-Ontology (GO) analysis showed that genes involved in pathways and processes related to the immune response, cell adhesion, and development were significantly hypo-methylated in SHC-MSCs (Fig. 2c). Consistently, GSEA indicated that cell adhesion-related gene sets, including the FAK pathway (NES = −2.09; FDR = 0.056) and the integrin pathway (NES = −1.73; FDR = 0.243), were significantly represented among unmethylated genes in SHC-MSCs (Supplementary Fig. 3a,b). We performed gene network (MetaCore) and leading-edge (GSEA) analyses to identify the driver genes. WNT-associated and MYC-associated gene networks were characteristically represented in SHC-MSCs (Supplementary Fig. 3c,d). Related biomarkers such as *FOG2* and *DHRS3* were significantly up-regulated in SHC-MSCs (Supplementary Fig. 4a), consistent with the increased level of DNA demethylation (Supplementary Fig. 4b). Furthermore, in comparison with naïve MSCs, SHC-MSCs exhibited the increased DNA demethylation in the pluripotency markers *OCT4* and *SALL4* (Supplementary Fig. 2f), as well as paternally imprinted *H19*, *RASGRF1*, and *DLK1*-*MEG3* loci (Supplementary Fig. 2g and Supplementary Fig. 5), which are characteristic epigenetic signatures of VSELs [20, 29], suggesting there is a relationship between SHC-MSCs and VSELs.

Next, we compared the expression profiles of naïve MSCs and SHC-MSCs, and identified 749 differentially expressed genes (500 up-regulated and 249 down-regulated) (Fig. 2d and Supplementary Fig. 6a). GO analysis of transcriptome data revealed that SHC-MSCs exhibited distinct expression of genes related to cell cycle processes (Supplementary Fig. 6b). Accordingly, SHC-MSCs demonstrated up-regulation of the MYC-associated gene network, involving *PLK1*, cell cycle-dependent kinase-1 (*CDK1*), *Cyclin B1*, and *DHRS3* (Fig. 2e), as well as the cAMP-responsive element-binding protein-1 (CREB1) gene network, involving chromosome-associated protein-C (*CAP-C*) and minichromosome maintenance-10 replication initiation factor (*MCM10*) (Supplementary Fig. 6c). Gene expression analysis showed that the majority of genes in the MYC and CREB1 gene networks, such as *ZNF143*, *PLK1*, *BIRC5*, and *CDC20*, were significantly up-regulated in SHC-MSCs (Fig. 2f).

To further identify genes underlying the enhanced properties of SHC-MSCs, we listed 68 genes that were hypo-methylated and whose expression was increased ≥1.5-fold in these cells (Fig. 2g,h, and Supplementary Fig. 7). mRNA expression of these genes was significantly up-regulated in SHC-MSCs (Fig. 2i). Moreover, protein expression of PLK1, ZNF143, FOG2, and DHRS3, which were highly ranked in the gene network and leading-edge analyses, was increased in SHC-MSCs (Fig. 2j).

### Overexpression of PLK1, ZNF143, FOG2, and DHRS3 induces primitive MSCs

**Fig. 3:**
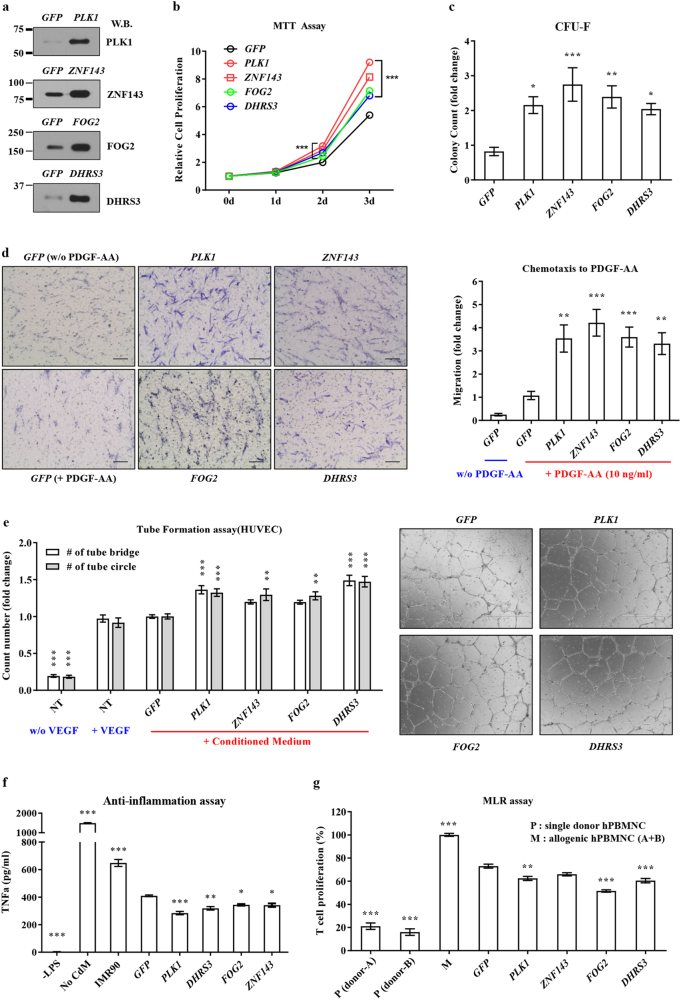
Overexpression of SHC-MSC biomarkers enhances the functions of MSCs (**a**) Western blot analysis of MSCs overexpressing *PLK1*, *ZNF143*, *FOG2*, and *DHRS3*. GFP was used as a control. (**b**–**d**) Enhanced proliferation (**b**, *n* = 12), CFU-F activity (**c**, *n* = 7), and chemotactic response to 10 ng/mL PDGF-AA (**d**, *n* = 7) of MSCs overexpressing these genes. Representative images of Transwell inserts from the chemotaxis assay are presented (×200 magnification, scale bar = 100 μm). **e**, **f** Representative examples (×40 magnification, scale bar = 1000 μm, right panel in **e**) and quantitative data (left panel in **e**) from the in vitro tube formation and anti-inflammatory assays (**f**, *n* = 4) using CM prepared from the indicated cells. Tube formation (*n* = 9) was quantified based on the number of bridges or circles. Quantitative data show the fold change relative to the control group. **g** Proliferation of human T-cells in the MLR assay (*n* = 6). PBMNCs were co-cultured with MSCs overexpressing the indicated open reading frame. The proliferation of responding cells is shown as a percentage relative to the positive control (M; set to 100%). Data are mean ± SEM. *p < 0.05, **p < 0.01, ***p < 0.001, one-way or two-way ANOVA with the Bonferroni post-hoc test

To explore the biological significance of the biomarkers of SHC-MSCs, we overexpressed *PLK1*, *ZNF143*, *FOG2*, and *DHRS3* in naïve MSCs (Fig. 3a) and examined the functions of these cells. MSCs expressing each of these genes had a significantly higher proliferation rate (Fig. 3b) and CFU-F activity (Fig. 3c and Supplementary Fig. 8a), an indicator of clonogenic progenitor cells, than control MSCs expressing green fluorescent protein (GFP). Moreover, MSCs overexpressing these genes exhibited a higher level of chemoattraction to platelet-derived growth factor (PDGF) (Fig. 3d), indicative of improved mobilization and homing. Furthermore, conditioned medium (CM) of MSCs overexpressing these genes had an improved angiogenic potency in the Matrigel tube formation assay (Fig. 3e and Supplementary Fig. 8b) and improved anti-inflammatory activity based on the significant inhibition of tumor necrosis factor (TNF)-α secretion by LPS-stimulated macrophages (Fig. 3f). When immunomodulatory properties were evaluated by the MLR assay, MSCs overexpressing these genes strongly repressed the proliferation of PBMNCs upon allogeneic stimulation (Fig. 3g), similar to SHC-MSCs (Fig. 1j). These in vitro functional assays demonstrate that overexpression of *PLK1*, *ZNF143*, *FOG2*, and *DHRS3* induces MSCs in a primitive state, as evidenced by their improved proliferative, self-renewal, migratory, pro-angiogenic, anti-inflammatory, and immunomodulatory capacities, which are crucial for therapeutic potency.

### SHC-MSCs have an enhanced potency for treating GVHD

**Fig. 4:**
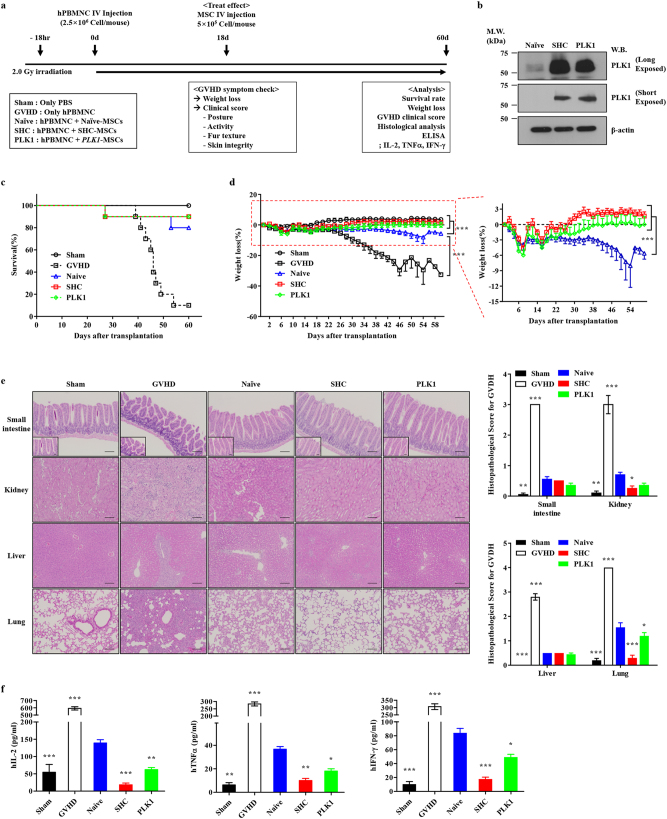
SHC-MSCs and *PLK1*-overexpressing MSCs demonstrate an increased efficacy for treating GVHD (**a**) Experimental scheme of MSC administration in a humanized GVHD mouse model. Mice were injected with 2.5 × 10^6^ human PBMNCs followed by 5 × 10^5^ naïve MSCs (Naïve group), SHC-MSCs (SHC group), or *PLK1*-overexpressing MSCs (PLK1 group). The Sham group was injected with PBS instead of PBMNCs. **b** Western analysis of PLK1 in the indicated MSCs prior to administration. **c**–**f** Survival rate (**c**, *n* = 10), body weight (**d**, *n* = 10), histological analysis of GVHD target organs (**e**, *n* = 10, left panel, ×200 magnification, scale bar = 100 μm), and plasma levels of the human inflammatory cytokines IL-2, TNF-α, and IFN-γ (**f**, *n* = 20) in mice from the indicated groups. Data are mean ± SEM. *p < 0.05, **p < 0.01, ***p < 0.001, one-way or two-way ANOVA with the Bonferroni post-hoc test

To examine the in vivo significance of the improved anti-inflammatory and immunosuppressive functions of SHC-MSCs, we injected these cells into a humanized GVHD mouse model transplanted with human PBMNCs [8] and compared the therapeutic outcomes with those achieved using naïve and *PLK1*-overexpressing MSCs (Fig. 4a,b). At 8 weeks after transplantation, about 90% of mice transplanted with human PBMNCs alone (GVHD group) died, while the majority of mice transplanted with naïve MSCs (80%), SHC-MSCs (90%), and *PLK1*-overexpressing MSCs (90%) survived (Fig. 4c). Mice in the GVHD group exhibited severe weight loss, which was attenuated by treatment with the various types of MSCs (Fig. 4d). GVHD mice injected with SHC-MSCs or *PLK1*-overexpressing MSCs exhibited better survival and less weight loss than those injected with naïve MSCs (Fig. 4c,d). Clinical scoring and histological analyses of representative GVHD target organs, including the small intestine, lung, kidney, and liver, revealed that injection of SHC-MSCs or *PLK1*-overexpressing MSCs effectively decreased immune cell infiltration and characteristic tissue injuries in GVHD mice, such as sloughing of villi in the small intestine and fibrosis in the lung and liver (Fig. 4e). Accordingly, tissue repair tended to be better in mice injected with SHC-MSCs or *PLK1*-overexpressing MSCs than in those injected with naïve MSCs (Fig. 4e). Furthermore, immunofluorescence staining of human β2-microglobin showed that homing and engraftment of SHC-MSCs and *PLK1*-overexpressing MSCs into these GVHD target organs were better than those of naïve MSCs (Supplementary Fig. 9). Consistent with the anti-inflammatory and immunomodulatory activities observed in vitro (Fig. 1, Fig. 3f,g), levels of human inflammatory cytokines, including IL-2, TNF-α, and interferon (IFN)-γ, in blood samples of GVHD mice were more effectively reduced by injection of SHC-MSCs or *PLK1-*overexpressing MSCs than by injection of naïve MSCs (Fig. 4f). Taken together, these findings demonstrate that the SHC procedure and *PLK1* overexpression enhance the therapeutic potency of MSCs for treating GVHD.

## Discussion

A number of preclinical and clinical trials employing MSCs are currently in progress worldwide. However, the clinical use of these cells is hampered by the lack of optimized methods to stabilize primitive MSCs in vitro [30, 31]. The present study describes a simple method to isolate and maintain primitive small SCs. SHC-MSCs exhibited enhanced functions in vitro and an improved potency for treating GVHD in vivo. We also demonstrated that the SHC procedure affects biological pathways related to immune modulation, cell adhesion, and the cell cycle by modulating expression of *PLK1*, *ZNF143*, *FOG*, and *DHRS3*. These findings indicate that the SHC procedure facilitates the in vitro isolation of primitive small MSCs. Such cells seem to be at the top of the SC hierarchy in adult tissues and likely play a role in tissue and organ regeneration.

MSCs isolated from BM, placenta, umbilical cord, and peripheral blood have been used to treat a range of cardiovascular, musculoskeletal, neurological, and immunological diseases, including GVHD, due to their many advantages. Specifically, these MSCs are easily harvested, are multipotent, can be expanded ex vivo, display little or no immunogenicity due to the lack of HLA-DR expression, lead to little tumorigenicity, and are associated with fewer ethical problems than human embryonic SCs [32]. MSC therapy effectively improved the clinical outcome of patients with the aforementioned intractable disorders in clinical trials [33–35]. However, MSCs are heterogeneous in terms of their morphology (small vs. flattened), expression of surface markers (CD133, CD44, CXCR4, CD49f, and CD146), and expression of pluripotency-associated transcription factors (*OCT4*, *NANOG*, and *SOX2*) (Fig. 1g and Supplementary Fig. 2c−f) due to a developmental hierarchy. Therefore, elucidation of the morphologic and molecular features of the most primitive MSCs can not only improve understanding of the developmental hierarchy of MSCs, but also help to develop an optimized method for isolating and maintaining such cells in vitro for therapeutic purposes [16].

Several independent research groups reported the presence of rare pluripotent or multipotent MSC-like populations in adult tissues including MSCs [36, 37], multipotent adult progenitor cells [38], marrow-isolated adult multilineage inducible cells [39], multipotent adult SCs [40], elutriation-derived (Fr25/Lin^−^) SCs [41–43], spore-like SCs [44], pluripotent Sca-1^+^CD45^−^c-kit^−^ cells [45], multilineage-differentiating stress-enduring SCs [46–48], and VSELs [49–52]. These cells are all small and express pluripotency-associated transcription factors. Our SHC procedure, which combines the beneficial effects of exposure to hypoxia and a high concentration of Ca^2+^, can enrich and expand primitive small SCs. Similar to the aforementioned small cells, SHC-MSCs expressed early development genes (*OCT4, NANOG, STELLA*, and *SALL4*) and functional surface markers (e.g., CXCR4, CD49f, and CD146) and exhibited an enhanced self-renewal capacity (Fig. 1g and Supplementary Fig. 2c−f). This suggests that these cells represent overlapping populations of primitive SCs in heterogeneous populations and have the potential to differentiate into cells of all three germ layers.

VSELs exhibit the following unique epigenetic features: i) an open/active chromatin structure in the *Oct4* promoter, ii) epigenetic changes of some imprinted genes that regulate insulin/insulin-like growth factor signaling (*Igf2*, *H19*, *Igf2R*, and *Rasgrf1*), and iii) DNA demethylation in the promoters of germline lineage genes [19, 20, 29]. These unique signatures ensure the tight regulation of pluripotency and quiescence in VSELs deposited in adult tissues. SHC-MSCs shared several molecular features with VSELs. In comparison with naïve MSCs, SHC-MSCs had an open/active chromatin structure in the *OCT4* promoter (Supplementary Fig. 2e,f) and demethylated DNA in *H19*, a paternally imprinted gene, and the promoter of *SALL4*, a germline lineage gene (Supplementary Fig. 2f,g). These results indicate there is a relationship between SHC-MSCs and VSELs. Thus, a further study comparing the molecular features of SHC-MSCs and small primitive SCs, including VSELs, is required not only to elucidate their developmental hierarchy, but also to identify biomarkers of small primitive SCs.

In terms of therapeutic applications, small MSCs can move through the lung microvasculature and be efficiently distributed to other tissues. Human MSCs cultured as 3D spheroids are smaller than those cultured in a standard monolayer. Moreover, a larger fraction of spheroid MSCs are recovered from the liver, spleen, kidney, and heart of NOD/severe compromised immunodeficient (SCID) mice when injected via the tail vein, with fewer trapped in lung tissue, based on an in vivo tracking assay [53]. TNF-α-stimulated gene/protein-6, stanniocalci-1, anti-inflammatory proteins, anti-apoptotic proteins, and CXCR4 are up-regulated in small MSCs cultured as 3D spheroids, leading to enhanced anti-inflammatory and tissue-regenerative effects with improved cell survival after transplantation [53, 54]. The survival of transplanted cells and their movement to damaged organs affect the efficacy of cell-based therapies and may be crucial for the treatment of GVHD, a multi-organ disorder characterized by immune dysfunction [3]. We previously reported a system for rapid clinical-scale expansion of UCB-MSCs involving exposure to a combination of Ca^2+^ and hypoxia, which synergistically promotes cell growth and expression of stemness genes, but delays cellular senescence [22]. To optimize the therapeutic efficacy of MSCs for GVHD, the SHC procedure was used to enrich small primitive MSCs, which have improved homing and engraftment capacities in injured tissues. Indeed, SHC-MSCs showed better homing and engraftment in several GVHD target organs than naïve MSCs (Supplementary Fig. 9). Furthermore, the enhanced anti-inflammatory and immunomodulatory effects of SHC-MSCs (Fig. 3) led to an improved therapeutic potency in GVHD (Fig. 4). Collectively, the SHC procedure can enrich small primitive MSCs with many advantages for treating GVHD and can be easily adapted for the clinical-scale expansion of several types of MSCs.

Mechanistically, our transcriptome and DNA methylome analyses demonstrated that genes related to immune modulation, cell adhesion, and the cell cycle were up-regulated in SHC-MSCs (Fig. 2). This correlated well with the enhanced proliferative, clonogenic, migratory, anti-inflammatory, and immunomodulatory activities of these cells (Fig. 1). These enhanced functions seemed to be associated with the SHC procedure, likely due in part to the induction of *PLK1*, *ZNF143*, *FOG2*, and *DHRS3* expression. Indeed, MSCs ectopically expressing these biomarkers demonstrated similar beneficial effects as SHC-MSCs (Fig. 3).

The enhanced immunomodulatory functions of SHC-MSCs and *PLK1*-overexpressing MSCs were confirmed in a humanized mouse model of GVHD (Fig. 4). Administration of these cells resulted in improved survival, less weight loss, and reduced histological evidence of GVHD (Fig. 4c,d). In the animal model of GVHD used in this study, effector cells were human PBMNCs infused into immunocompromised mice. These mice were generated by introducing an IL-2 receptor gamma mutation into mice with a NOD-SCID background (NOD-SCID IL-2rγ^null^; NSG mice). The resultant mice exhibit reduced activities of T-cells, B-cells, and NK cells, leading to engraftment of high levels of human PBMNCs [55]. To recapitulate the clinical setting in this humanized mouse model of GVHD, PBMNCs freshly isolated from healthy donors were used, mice were preconditioned by exposure to 2.0 Gy irradiation prior to PBMNC infusion, and human MSCs allogeneic to the PBMNC donor were intravenously injected at 18 days after PBMNC transfusion (Fig. 4a). Thus, this GVHD model is a reliable system to evaluate the effects of batches of clinical MSC therapeutics against donor lymphocytes. However, the immune reaction in a xenogeneic setting may be distinct to the allogenic response in human GVHD patients. An in vitro MLR assay demonstrated that SHC-MSCs and *PLK1*-overexpressing MSCs had enhanced immunomodulatory activities during the allogeneic response. However, further studies concerning the clinical relevance and mode of action are required to successfully translate the promising preclinical results from this xenograft model into clinical practice.

GVHD is a leading cause of late morbidity and mortality after allogeneic hematopoietic SC transplantation. However, there is a lack of effective therapies, no standards of care for second-line therapy, and dependence on steroids for first-line therapy [3]. MSC-based therapies are being developed and yielding exciting results [4, 5]. However, it is difficult to adapt MSCs as a first-line treatment for established GVHD due to the high cost and lack of a standardized preparation procedure [30, 31]. Therefore, *ex vivo* expansion of functionally qualified MSCs using a cost-effective and safe (without genetic manipulation) procedure, such as SHC, may be the optimal strategy to improve the results of future clinical trials of MSCs to treat immune-related disorders.

In summary, we demonstrated that the SHC procedure is a simple and reliable method to isolate small primitive SCs with enhanced self-renewal and immunomodulatory capacities. This will accelerate the clinical use of MSCs to treat conditions such as GVHD, the incidence of which exceeds 50% among transplant patients and whose treatment is hampered by the lack of therapeutic strategies. Furthermore, our findings can be applied to various conditions associated with immune deregulation such as autoimmune diseases. By establishing useful guidelines for the collection and expansion of primitive MSCs, this study will facilitate investigations of the molecular mechanisms underlying the dynamics of repopulating adult SCs in normal physiological and pathological conditions to improve understanding of the SC hierarchy in adult tissues.

## Electronic supplementary material

Supplementary Information
