# Supplementary material for: Small hypoxia-primed mesenchymal stem cells attenuate graft-versus-host disease
Source: Leukemia. 2018 May 22;32(12):2672–84. doi: 10.1038/s41375-018-0151-8 (PMC6286327; doi:10.1038/s41375-018-0151-8)
Supplement: Supplementary file 1 — Supplementary Information [file 41375_2018_151_MOESM1_ESM.docx]

**Small hypoxia-primed mesenchymal stem cells attenuate graft-versus-host disease**

YongHwan Kim^1,2,§^, Hye Jin Jin^3,§^, Jinbeom Heo^1,2,§^, Hyein Ju^1,2^, Hye-Yeon Lee^1,2^, Sujin Kim^1,2^, Seungun Lee^1,2^, Jisun Lim^1,2^, Sang Young Jeong^3^, JiHye Kwon^3^, Miyeon Kim^3^, Soo Jin Choi^3^, Wonil Oh^3^, Yoon Sun Yang^3^, Hyun Ho Hwang^4^, Hwan Yeul Yu^1,2^, Chae-Min Ryu^1,2^, Hong Bae Jeon^3,*^ Dong-Myung Shin^1,2,*^

^§^These authors equally contributed to this work.

**Running title:** Improved treatment of GVHD using SHC-MSCs

**^*^Corresponding authors:**

Dong-Myung Shin, Ph.D.

Department of Biomedical Sciences, Asan Medical Center, University of Ulsan College of Medicine, Pungnap-2 dong, Songpa-gu, Seoul, 05505, Korea

Tel: 82-2-3010-2086; Fax: 82-2-3010-8493; Email: d0shin03@amc.seoul.kr

Hong Bae Jeon, Ph.D.

Biomedical Research Institute, MEDIPOST Co., Ltd., Seongnam, 13494, Korea

Tel: 82-2-3465-6772; Fax: 82-2-3465-6754; Email: jhb@medi-post.co.kr

**This PDF file contains :**

**Nine Supplemental Figures (Figure S1 − S9) and Supplementary Figure Legend**

**One Supplementary Table**

**Supplementary Method and References**

**SUPPLEMENTARY FIGURE LEGENDS**


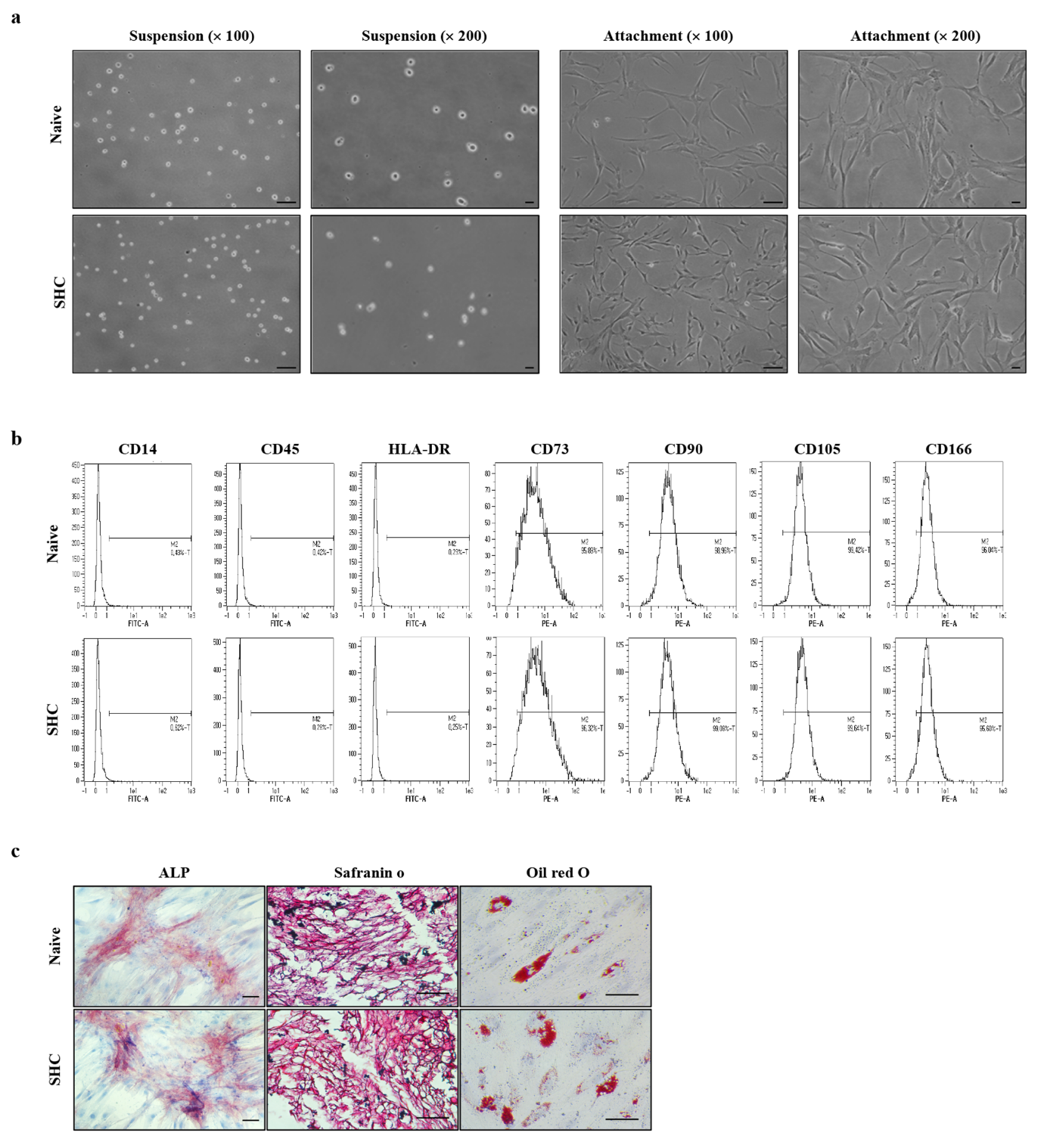


**Supplementary Fig. S1. Immunophenotype and multilineage differentiation of SHC-MSCs**

**(a)** Representative microscopic images of naïve MSCs and SHC-MSCs (×100 magnification; scale bar=50 μm) in suspension or adhered to a tissue culture dish (attachment). Higher magnification images (×200, scale bar=10 μm) are shown on the right. **(b)** The immunophenotype of naïve MSCs and SHC-MSCs was examined by flow cytometry. **(c)** The multilineage potential of naïve MSCs and SHC-MSCs was analyzed by alkaline phosphatase (ALP, for osteogenesis), Safranin O (for chondrogenesis), and Oil Red O (for adipogenesis) staining (×200 magnification, scale bar=50 μm).

**
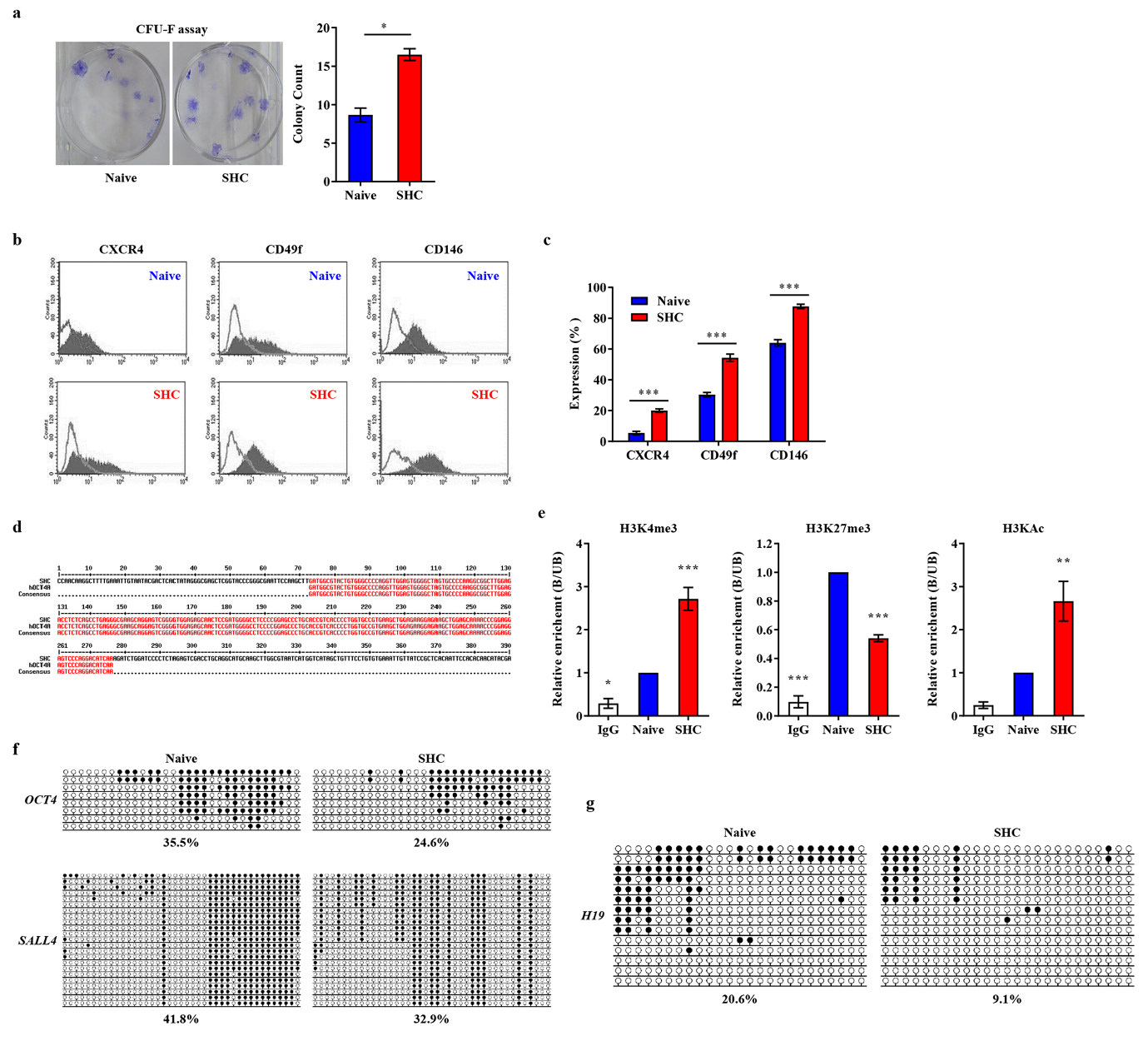
**

**Supplementary Fig. S2. Enhanced self-renewal capacities of SHC-MSCs**

**(a − c)** CFU-F assay **(a)**, flow cytometry analysis (**b**) and quantification of expression of surface markers of primitive MSCs (CXCR4, CD49f, and CD146) (**c**) in naïve and SHC-MSCs at P5. Quantitative data are shown as mean ± SEM (n=3). *p<0.05, ***p<0.001, Mann–Whitney *U* test or two-way ANOVA with the Bonferroni post-test. **(d)** Sequencing results of the PCR amplicons for detecting human *OCT4* expression. Sequencing data was aligned with the sequences for the exon 1 and exon 2 of human *OCT4A* transcript. **(e)** qChIP analysis for acetylated histone H3 (H3Ac) and trimethylation of lysine 4 (H3K4me3) or trimethylation of lysine 27 (H3K27me3) on the histone H3 protein subunit in naïve or SHC-MSCs. Enrichment of the indicated histone modifications was calculated as the ratio of the value of the bound fraction (B) to that of the unbound fraction (UB). The fold difference is represented by the ratio to naïve MSCs (set to 1) and is displayed as means ± SEM (n=4) (*p<0.05, **p<0.01, ***p<0.001, one-way ANOVA). **(f and g)** Bisulfite sequencing of the pluripotency or germline genes **(f)** and *H19* paternally imprinted genes **(g)** is shown for the indicated cells. The numbers under each BSS profile indicate the percentage of methylated CpG sites. Methylated and unmethylated CpG sites in bisulfite sequences are shown as filled and open circles, respectively.


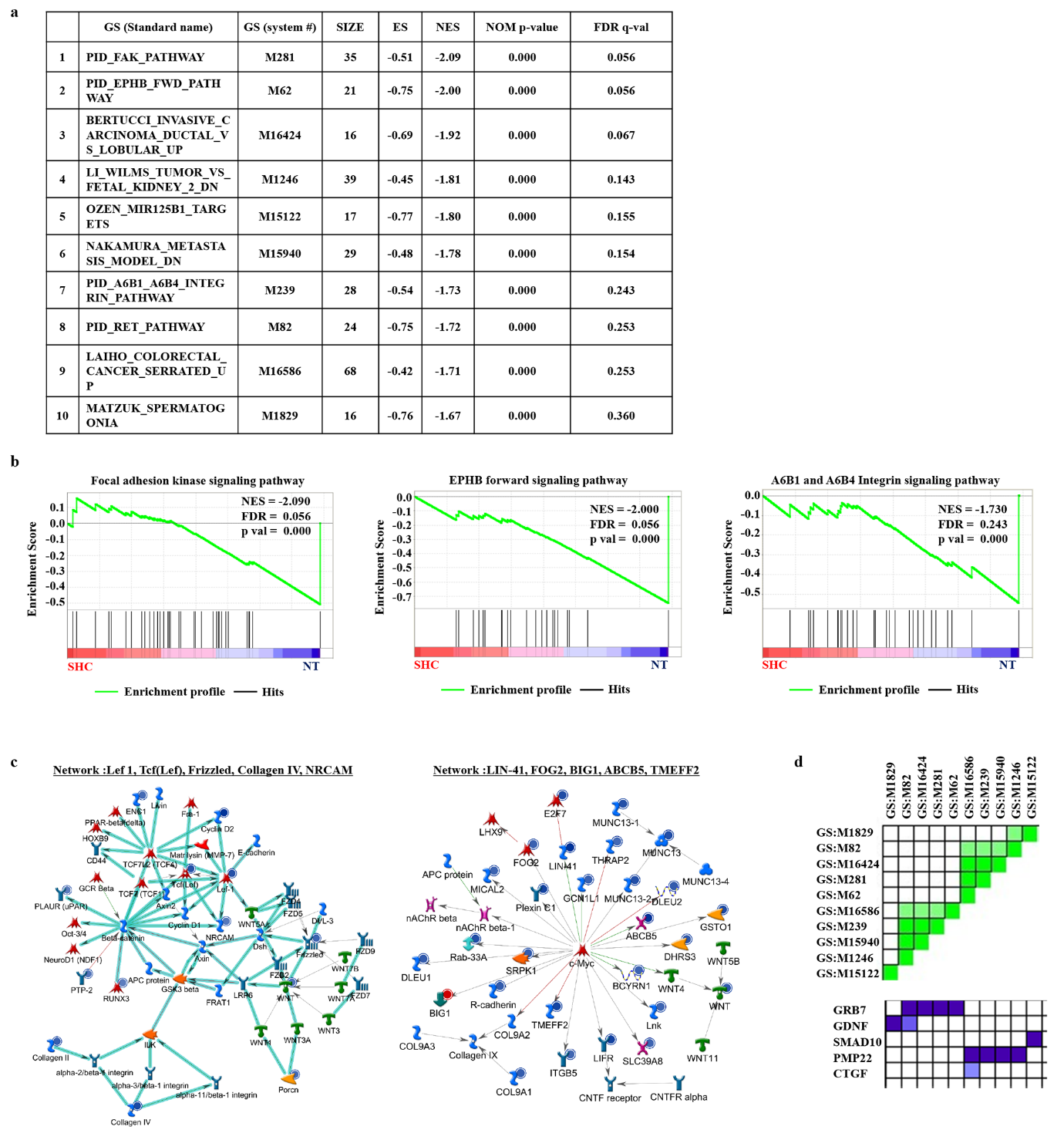


**Supplementary Fig. S3. Gene sets that are hypo-methylated in SHC-MSCs**

**(a and b)** The top-scoring 10 gene sets **(a)** and three representative ES plots **(b)** in GSEA analysis on comparison between SHC-MSCs and naïve (NT) MSCs. The genesets were listed according to the normalized enrichment score (NES). GSEA; gene set enrichment analysis, ES; enrichment score, NOM p-val; nominal p value, FDR; false discovery rate. **(c and d)** Two representative *WNT-*, *FOG2-*, *DHRS3*-associated gene networks **(c)** identified via MetaCore and leading-edge subset analyses (top panel in **d**) and the corresponding target genes (lower panel in **d**). The top 10 hypo-methylated gene sets (**Supplementary Fig. S3a**) in the comparison of the DNA methylation profiles of naïve MSCs and SHC-MSCs were used. Gene networks are illustrated by overlaying delta_mean values in SHC-MSCs versus naïve MSCs. Hyper- and hypo-methylated genes are indicated in red and blue, respectively.


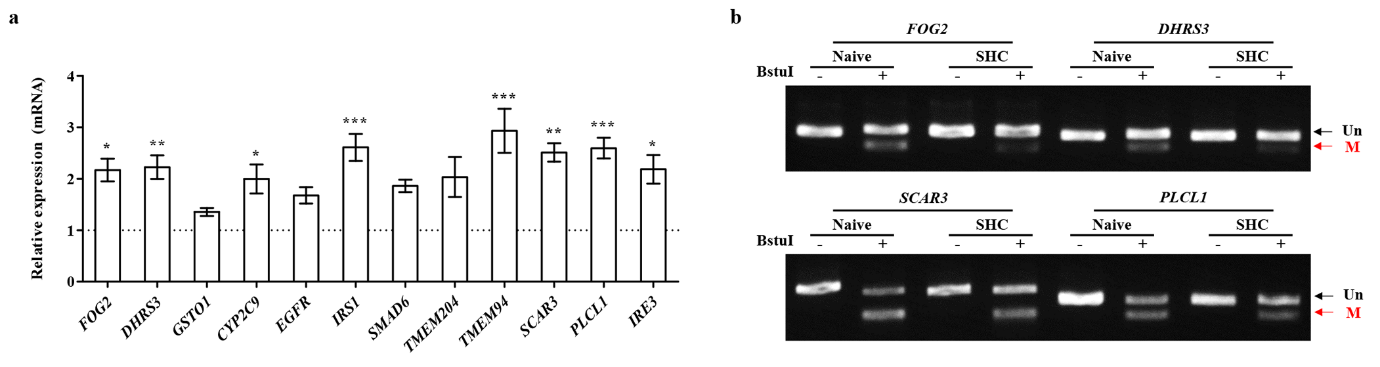


**Supplementary Fig. S4. Up-regulation of *FOG2* and *DHRS3* in SHC-MSCs**

**(a)** Real-time qPCR analysis of hypo-methylated genes in the *WNT-*, *FOG2-*, *DHRS3*-associated gene networks. Expression in SHC-MSCs is shown relative to that in naïve MSCs (set to 1; indicated by the red dotted line). Data are mean ± SEM (n=4). *p<0.05, **p<0.01, ***p<0.001, one-way ANOVA with the Bonferroni post-test. **(b)** Combined bisulfite restriction analysis of the indicated genes in naïve MSCs and SHC-MSCs. Un, unmethylated DNA; M, methylated DNA.


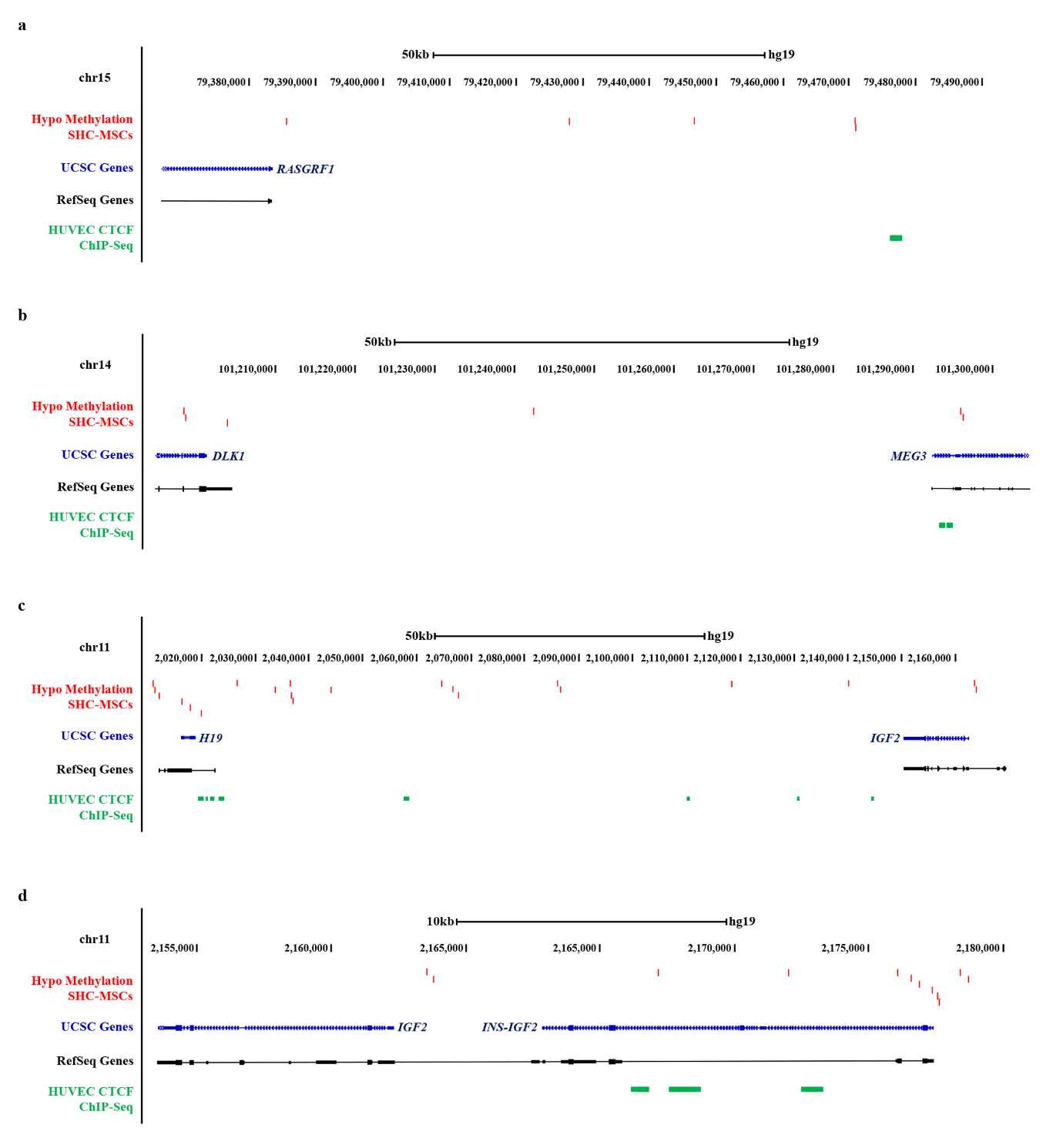


**Supplementary Fig. S5. DNA methylation status of paternally imprinted loci in SHC-MSCs**

**(a − d)** Snapshots of the UCSC genome browser for *RASGRF1* **(a)**, *DLK1*-*MEG3* **(b)**, and *H19*-*IGF2* **(c)** paternally imprinted loci, and *INS* locus **(d)** marked with the regions hypomethylated in SHC-MSCs as red lines. DNA hypomethylation were determined using delta_mean ≤ -0.1 (the difference of methylation signal, avg beta of SHC-MSC – avg beta of naïve MSC). The snapshots of the UCSC genome browser were aligned with the CTCF ChIP-seq database (in HUVEC cells) of the ENCODE project marked as green lines. Exons of each gene in UCSC or RefSeq genes are shown in boxes. *RASGRF1*, Ras protein specific guanine nucleotide releasing factor 1*; DLK1*, delta like non-canonical Notch ligand 1; *MEG3*, maternally expressed 3; *IGF2*, insulin like growth factor 2; INS, insulin; CTCF, CCCTC-binding factor; HUVEC, human umbilical vein endothelial cells.


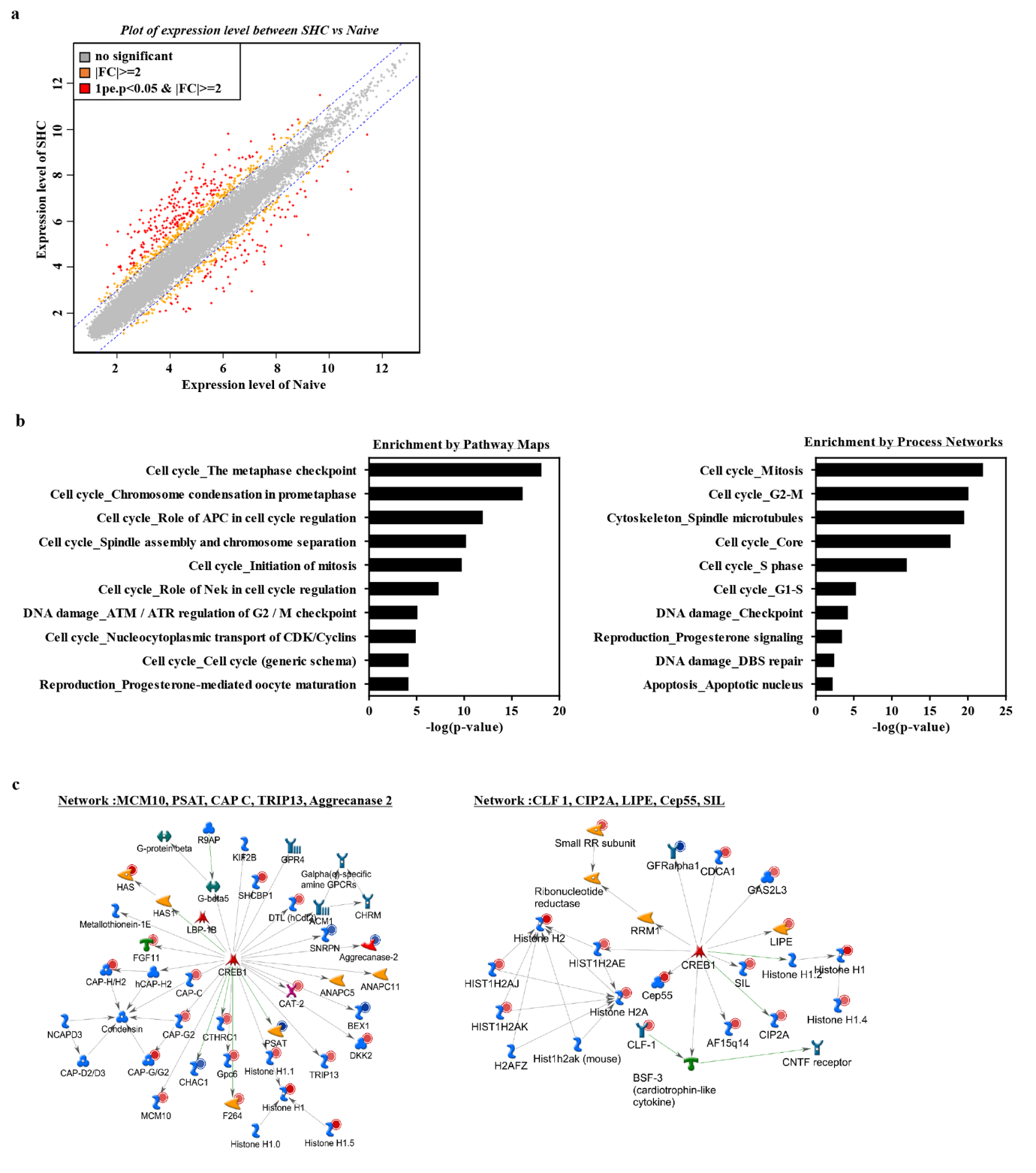


**Supplementary Fig. S6. Transcriptome analysis characteristic to SHC-MSCs**

**(a − c)** Scatter plot **(a)** of genes differentially expressed between naïve and SHC-MSCs and related Gene Ontology **(b)** and gene network **(c)** analysis using Metacore assay. Gene networks are illustrated by overlaying experimental values as fold changes (FC) for the SHC vs naïve comparison. Up- and downregulated genes are indicated in red and blue, respectively.

**
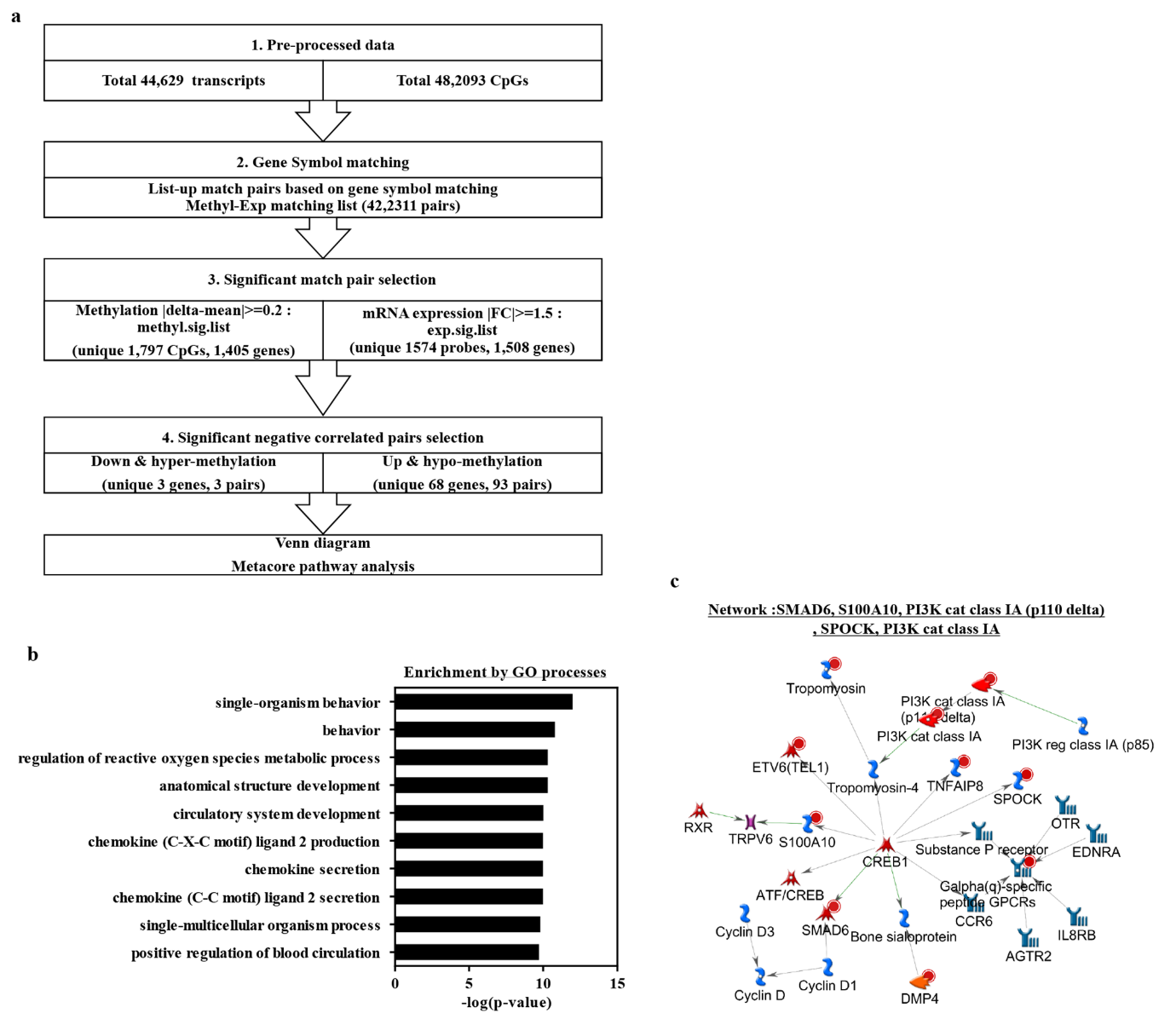
**

**Supplementary Fig. S7. Multi-omic analysis characteristic to SHC-MSCs**

**(a)** Summary of the multi-omic analysis for DNA methylome and transcriptome datasets to identify the genes which hypo-methylated and at the same time increased expression (≥ 1.5 folds) in SHC-MSCs, compared with naïve cells. **(b and c)** The selected 68 genes were used for Gene Ontology **(b)** and gene network **(c)** analysis using Metacore assay. Up- and downregulated genes are indicated in red and blue, respectively


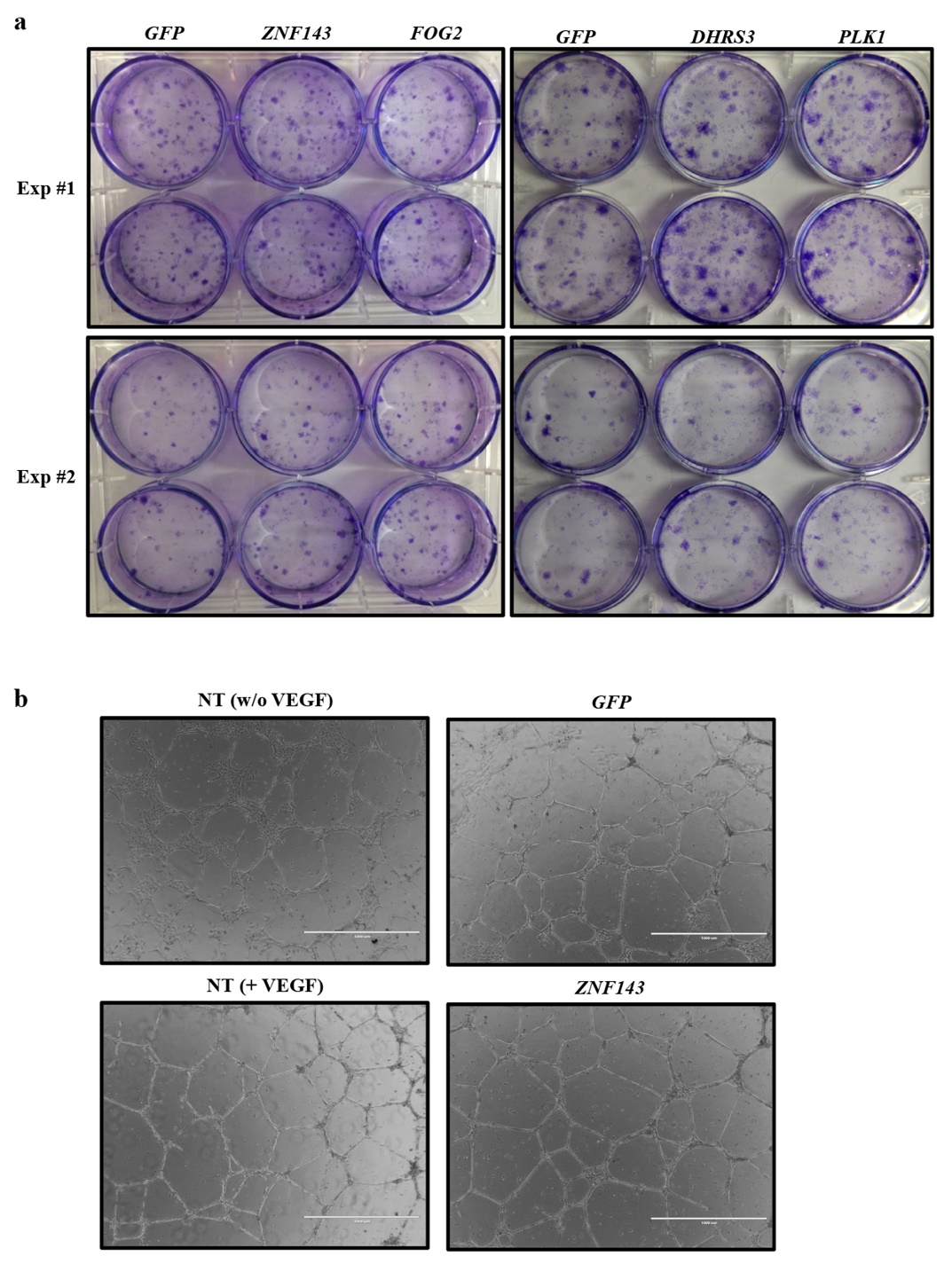


**Supplementary Fig. S8. Enhanced core stem cell functions of SHC-MSCs**

**(a and b)** Representative data from the CFU-F **(a)** and *in vitro* tube formation **(b)** assays using MSCs expressing *PLK1*, *ZNF143*, *FOG2*, *DHRS3*, or GFP. In the CFU-F assay, cells were seeded into 6-well plates at a density of 60 cells/well and cultured for 14 days, and then the number of colonies was counted. For the *in vitro* tube formation assay, CM was prepared from the indicated MSCs. Saline and recombinant human VEGF-A (50 ng/mL) were used as negative and positive controls, respectively (×40 magnification, scale bar=1000 μm).

**
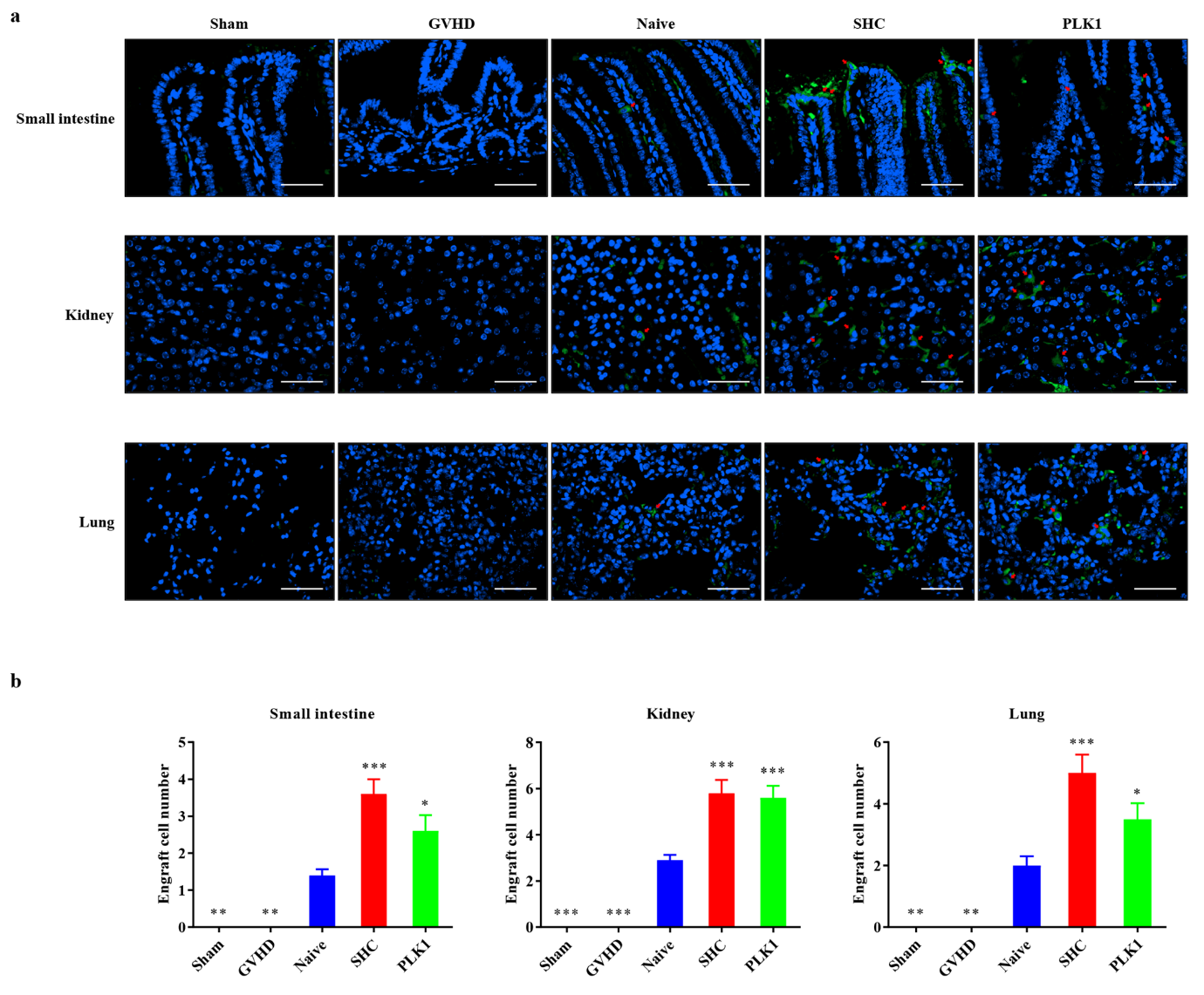
**

**Supplementary Fig. S9. Immunostaining analysis for the engraftment of SHC-MSC.**

**(a)** Representative fluorescent microscopic images of small intestine, kidney, and lung tissue sections of a humanized GVHD mice after immuno-staining for human β2-microglobulin expressing cells (green, ×400 magnification, scale bar=50 μm). Nuclei were stained with DAPI (blue). **(b)** Quantification of the engrafted human cells (marked with red arrows, n=10) in mice from the indicated groups. Data are mean ± SEM. *p<0.05, **p<0.01, ***p<0.001, one-way ANOVA with the Bonferroni post-hoc test.

**Supplementary Table 1. Basic information regarding the hUCB-MSCs used in this study.**

| **hUCB-MSCs** | **Maternity (age)** | **Cell Surface marker** | | **Differentiation** |
| --- | --- | --- | --- | --- |
|  |  | **Positive** | **Negative** |  |
| **#1** | **33** | **Pass** | **Pass** | **Pass** |
| **#2** | **35** | **Pass** | **Pass** | **Pass** |
| **#3** | **30** | **Pass** | **Pass** | **Pass** |
| **#4** | **38** | **Pass** | **Pass** | **Pass** |
| **#5** | **32** | **Pass** | **Pass** | **Pass** |

hUCB-MSCs were harvested from 5 independent donors (hUCB #1 to 5). MSC features were analyzed by representative MSC marker expression (Positive: CD29, CD73, CD90, CD105, CD166 ≥ 85%; Negative: CD14, CD45 ≤ 1.5%) or their capacity for differentiation (osteogenic, chondrogenic, and adipogenic dfferentiation).

**SUPPLEMENTARY METHODS**

**In vitro characterization of MSCs**

Senescence, proliferation, colony-forming unit-fibroblast (CFU-F) activity, surface marker expression, multipotency (*in vitro* differentiation into osteogenic, chondrogenic, and adipogenic lineages), and migration of naïve MSCs, SHC-MSCs, and MSCs overexpressing each of the aforementioned ORFs were assessed as previously described^1-3^.

**In vitro assay of anti-inflammatory activity**

The rat alveolar macrophage cell line NR8383 (ATCC, Rockville, MD) was cultured in Roswell Park Memorial Institute medium supplemented with 10% fetal bovine serum. NR8383 cells were activated with lipopolysaccharide (LPS) (1 μg/mL; Sigma) as a positive control. LPS-stimulated NR8383 cells were co-cultured with MSCs for 3 days^4^. Levels of rat IL-6 and IL-8 and human angiopoitin-1 (ANG-1) and vascular epidermal growth factor (VEGF) were measured in the culture medium using enzyme-linked immunosorbent assays (R&D Systems, Minneapolis, MN).

**Mixed lymphocyte reaction (MLR) assay**

Stimulator human peripheral blood mononuclear cells (PBMNCs; AllCells, LLC, Alameda, CA) and the indicated MSCs were inactivated by treatment with 10 μg/mL mitomycin-C (Sigma-Aldrich) for 1 h at 37°C. Inactivated PBMNCs (1 × 10^5^) or MSCs (1 × 10^4^) were added to each well of a 96-well culture plate. Responder PBMNCs (1 × 10^5^) from single-cell suspensions were added to the stimulator cells and incubated for 5 days at 37°C in 5% CO_2_. Alloantigen-induced proliferation was measured using the bromodeoxyuridine incorporation assay (Roche, Mannheim, Germany)^5^. The level of human PGE2 in mixed lymphocyte reaction (MLR) supernatants was analyzed using an enzyme-linked immunosorbent assay (R&D Systems).

**Transcriptome** **microarray hybridization and data processing**

Total RNA from various cells was isolated using the RNeasy Mini Kit (QIAGEN, Valencia, CA, USA), including treatment with DNase I (QIAGEN). One microgram of total RNA was subjected to analysis using the Affymetrix GeneChip Human (for hESC-derived M-MSCs) 2.0 ST Array (Affymetrix, Santa Clara, CA, USA). Microarray image data were processed on a GeneChip GCS3000 Scanner and Command Console software (Affymetrix). After importing CEL files of six samples (three independent samples from each group), the data were summarized and normalized using the robust multi-average (RMA) method implemented in Affymetrix Expression Console Software.

**DNA methylome analysis**

The DNA methylome of naïve and SHC mesenchymal stem cells (MSCs) was analyzed using on a Infinium HumanMethylation450K BeadChip assay (Illumina Inc, San Diego, CA). Genomic DNA (gDNA) were isolated using the DNeasy Blood & Tissue Kit (Qiagen Inc, Valencia, CA, USA). Next, 500 ng of gDNA were used in bisulfite conversion with Zymo EZ DNA methylation kit (Zymo Research, Irvine, CA). For sample amplification and hybridization for BeadChips, 200ng of input bisulfite-converted DNA (MA1) was required to create a sufficient quantity of DNA (1000 × amplification) on a single BeadChip in the infinium methylation assay (Illumina RPM and MSM)^6,7^. After amplification, the product was fragmented using a proprietary reagent (FMS), precipitated with 2-propanol (plus precipitating reagent; PM1), and resuspended in formamide-containing hybridization buffer (RA1). The DNA samples are denatured at 95°C for 20 min, then placed in a humidified container for a minimum of 16 h at 48 °C allowing CpG loci to hybridize to the 50 mer capture probes.

Following hybridization, the BeadChip/Te-Flow chamber assembly was placed on the temperature-controlled Tecan Flowthrough Chamber Rack, and all subsequent washing, extension, and staining were performed by addition of reagents to the Te-Flow chamber. For the allele specific single-base extension assay, primers were extended with a polymerase and labeled nucleotide mix (TEM), and stained with repeated application of STM (staining reagent) and ATM (anti-staining reagent). After staining was complete, the slides were washed with low salt wash buffer (PB1), immediately coated with XC4, and then imaged on the Illumina iScan Reader which is a two-color (532 nm/658 nm) confocal fluorescent scanner with 0.53 μm pixel resolution. The image intensities are extracted using Illumina’s iScan Control software.

For raw data preparation and Statistical analysis, the quality of hybridization and overall chip performance were monitored by visual inspection of both internal quality control checks and the raw scanned data. Raw data were extracted as beta values for each CpG for each sample using R watermelon package. Beta values were calculated by subtracting background using negative controls on the array and taking the ratio of the methylated signal intensity against the sum of both methylated and unmethylated signals. A beta value of 0.0-1.0 was reported significant percent methylation, from 0% to 100%, respectively, for each CpG site^8-10^. Array CpG probes that have detection p-value ≥ 0.05 (similar to signal to noise) in over 25% samples were filtered out. And then filtered data was background correction & dye bias equalization by R methylumi & lumi package. To reduce Infinium I and Infinium II assay bias, corrected signal value was normalized by BMIQ (Beta Mixture Quantile) method^11^. Differentially expressed methylation list were determined using |delta_mean| ≥ 0.2 (the difference of methylation signal, avg beta of Case – avg beta of Control) and p-value < 0.05 of independent t-test in which the null hypothesis was that no difference exists among 2 groups. All data analysis and visualization of differentially expressed genes was conducted using R 3.0.2 ([www.r-project.org](http://www.r-project.org)).

**Genome-wide gene expression and DNA methylation analyses**

Functional analyses of the transcriptome and DNA methylome databases for gene networks, biofunctions, and canonical pathways were performed using MetaCore microarray software (Clarivate Analytics, Philadelphia, PA) or gene set enrichment analysis (GSEA; Broad Institute, Cambridge, MA) with default settings. In MetaCore analysis, genes up- or down-regulated ≥1.5-fold with p<0.05 were defined as significantly changed. For GSEA analysis, gene sets were obtained from published literature or filtered from a curated functional gene set (C2) database, as previously described^12^. Significant differences were determined based on a false discovery rate (FDR) of <0.25.

**Real-time quantitative-polymerase chain reaction (RQ-PCR) and DNA methylation analyses**

The DNA methylation status at individual loci was investigated via combined bisulfite restriction analysis and bisulfite sequencing, as previously described^13,14^.

For RQ-PCR analysis, total RNA was isolated using a RNeasy Mini Kit (Qiagen Inc., Valencia, CA), which removes genomic DNA using DNase I. cDNA library construction and RQ‑PCR were performed using the PikoReal System (Thermo Scientific, Pittsburgh, PA) and iQ™ SYBR Green PCR Master Mix (Bio Rad, Hercules, CA), as previously described^15^. Primers used to detect *OCT4* recognized *OCT4A*, a pluripotency-specific transcript, but not variants unrelated to pluripotency such as *OCT4B^16^*. These primers targeted exon 1 (forward primer) and the exon 1-exon 2 junction (reverse primer) of *OCT4A* mRNA. All primer sequences used in DNA methylation and RQ-PCR assays are available on request.

**Chromatin-immunoprecipitation (ChIP) assay**

ChIP analysis was performed using a Magna ChIP G kit (Millipore, Billerica, MA) according to the manufacturer’s instructions. Cross-linked chromatin isolated from cell extracts (1×10^7^ cells) was fragmented in 500 μL of Nuclear Lysis Buffer using a Bioruptor Plus sonication device (Diagenode Inc, Denville, NJ) with standard settings (four 20-second pulses separated by a 30-second interval on ice). Fragmented chromatin was then immunoprecipitated using Protein G magnetic beads conjugated with 3 μg of ChIP-grade antibodies against acetylated histone H3 (Millipore), trimethylated lysine-4 of histone H3 (Abcam, Cambridge, MA), or trimethylated lysine-27 of histone H3 (Millipore), or with rabbit or mouse immunoglobulin G (IgG) control antibodies (Sigma-Aldrich). The enrichment of each histone modification was calculated as the ratio of bound to unbound amplicon fractions and represented as the mean ± SEM of four independent experiments. All primers used in the ChIP assay are available upon request.

**Immunostaining**

To detect OCT4 protein, MSCs were fixed with 4% paraformaldehyde (Sigma-Aldrich) for 5 min, stained with an anti-OCT4 mouse IgG monoclonal antibody (clone #7F9.2, Millipore), and then labeled with an Alexa 488-conjugated anti-mouse antibody (Molecular Probes, Grand Island, NY). Nuclei were counterstained with Hoechst 33342 (Invitrogen, Waltham, MA). Images were acquired using a ZEISS LSM800 confocal microscope system (Carl Zeiss, Munich, Germany).

**Western blot analysis**

Cell extracts (30 μg) were prepared in RIPA lysis buffer (Santa Cruz Biotechnology) supplemented with the protease and phosphatase inhibitor cocktails (Roche, Indianapolis, IN) and 2.5 mM NaB (Upstate-Millipore). After separated on 12% SDS-PAGE gels, the expression level of the indicated proteins was assessed by probing with monoclonal antibodies specific to p21^CIP1^ (#3688, Cell Signaling Technology, Danvers, MA), p16^INK4a^ (#81278, Abcam), PLK1 (sc-17783, Santa Cruz Biotechnology), FOG2 (sc-4436), ZNF143 (YF-MA11021, Abfrontier, Seoul, Korea), DHRS3 (#95297, Abcam, Cambridge, MA), and β-actin (A5441; Sigma-Aldrich). To quantify the density of the indicated protein bands, quantitative digital image analysis was performed using ImageJ software (National Institute of Mental Health, Bethesda, MD). Relative protein expression was calculated by normalization to β-actin.

**Ectopic expression and RNA interference (RNAi)**

To overexpress the four key proteins responsible for the beneficial effects of SHC procedures, human *PLK1* (OHS6085-213573881), *ZNF143* (MHS6278-202832687), *FOG2* (OHS1770-202319315), and *DHRS3* (OHS1770-202315600) open reading frame clones (Dharmacon Inc., Lafayette, CO) were sub-cloned into the pENTR4 entry vector (Invitrogen) and finally transferred into cloned into the pLenti7.5/V3-DEST lentiviral vector (Invitrogen) using the Gateway® Technology reaction (Invitrogen). Lentivirus was produced using a four-plasmid transfection system (Invitrogen). Two days after transfection into the 293 FT packaging cell line, supernatants containing recombinant pseudo-lentiviral particles were collected and concentrated by precipitation using Lenti-X concentrator (Clontech, Mountain View, CA). The concentrated virus was infected into naïve umbilical cord blood derived MSCs using 6 μg/mL polybrene (Invitrogen), and the infected cells were selected using 1 μg/mL puromycin (Invitrogen). The effects of ectopic expression were examined using RQ-PCR and western blot analysis.

**Histopathological analysis and scoring for GVHD animals**

Sixty days after intravenous injection of human peripheral blood mononuclear cells (hPB-MNCs), target organs (lung, liver, kidney, small intestine) were harvested from mice in all groups and fixed in 4% paraformaldehyde (PFA). After 24 hours of fixation, each organ was embedded in paraffin, sectioned to 5 μm using a microtome, and stained with hematoxylin and eosin (H&E). Histological score of GVHD in target tissues was scored base on level of lymphocyte infiltration. The score of lymphocyte infiltrates was determined as follow: 0, normal; 0.5, focal and rare; 1, focal and mild; 2, diffuse and mild; 3, diffuse and moderate; 4, diffuse and severe. Lymphocyte infiltration was observed at a magnification of ×200 with the use of an inverted microscope (EVOS XL Core Cell Imaging System, Life Technologies). Randomly chosen areas from each slide using ten independent animals per treatment group was used to quantify the digital image.

**ELISA assay**

To examine the level of cytokines including human interferon-γ (IFN-γ), tumor necrosis factor-α (TNF-α), and interleukin-2 (IL-2), plasma samples were prepared from circulating whole blood in all sacrificed GVHD mice, as previously described^17^. The plasma samples were analyzed by following ELISA kits; human IFN-γ (#550612), TNF-α (#550610), and IL-2 (#550611) (BD Biosciences Pharmingen, San Jose, CA). Quantitative data were from duplicate ELISA assays (n=10) from randomly selected five animals per group.

**Study reagents**

| **Material** | **Vendor** | **Cat. No.** |
| --- | --- | --- |
| MEM ɑ medium | Gibco | 12571-063 |
| RPMI 1640 medium | Gibco | 22400-089 |
| FBS | Hyclone | SH30919.03 |
| High-glucose DMEM | Gibco | 11668-019 |
| Dexamethasone | Sigma | D2915 |
| Beta-glycerol phosphate | Sigma | G9422 |
| L-ascorbic acid 2 - phosphate | Sigma | A8960 |
| Sodium pyruvate | Sigma | P8574 |
| L-proline | Sigma | P5607 |
| Transforming growth factor β3 | Sigma | T5425 |
| Bone morphogenic protein 6 | R&D System | 507-BP |
| Lipopolysaccharide | Sigma | L6529-1mg |
| Rat Interleukin 6 ELISA | R&D System | DY506 |
| Rat Interleukin 8 ELISA | R&D System | DY515 |
| Human Angiopoietin-1 ELISA | R&D System | DANG10 |
| Human Vascular epidermal growth factor ELISA | R&D System | DVE00 |
| Human Prostaglandin E2 ELISA | R&D System | KGE004b |
| Human MCP-1/CCL2 ELISA | R&D System | DCP00 |
| ITS premix | Becton Dickinson | 354352 |
| Isobutyl-1-methylxanthine | Sigma | I7018 |
| Insulin | Sigma | I7378 |
| ALP staining kit | Sigma | B5655 |
| Silver nitrate | Sigma | 209139 |
| Safranin O | Sigma | S8884 |
| Oil red O | Sigma | O0625 |
| Mitomycin-C | Sigma | L4287 |
| Bromodeoxyuridine | Roche | 11 647 229 001 |
| BrdU ELISA | Roche | 11 647 229 001 |
| Calcium chloride solution | Sigma | 21115-100ml |
| Anti-p16 INK4a | abcam | ab81278 |
| Anti-p21 Waf1/Cip1 | Cell signaling | 2947 |
| β-actin | Novousbio | NB600-501 |
| Anti-CD49f | BD Pharmingen | 562473 |
| Anti-CD146 | BD Pharmingen | 560846 |
| Anti-CXCR4 | BD Pharmingen | 555974 |
| Anti-H3Ac | Millipore | 06-599 |
| Anti-H3K27me3 | Millipore | 07-449 |
| Anti-H3K4me3 | Abcam | ab8580 |
| Anti-OCT4 | Millipore | MAB4419 |
| Senescence β-Galactosidase staining kit | Cell signaling | 9860 |
| PluriStrainer 10㎛ | PluriSelect | 43-50010-03 |

**SUPPLEMENTARY REFERENCES**

1 Kang H, Kim K-H, Lim J, Kim Y-S, Heo J, Choi J *et al.* The Therapeutic Effects of Human Mesenchymal Stem Cells Primed with Sphingosine-1 Phosphate on Pulmonary Artery Hypertension. *Stem Cells and Development* 2015; **24:** 1658-1671.

2 Jin HJ, Lee HJ, Heo J, Lim J, Kim M, Kim MK *et al.* Senescence-Associated MCP-1 Secretion Is Dependent on a Decline in BMI1 in Human Mesenchymal Stromal Cells. *Antioxid Redox Signal* 2016; **24:** 471-485.

3 Lim J, Kim Y, Heo J, Kim K-H, Lee S, Lee SW *et al.* Priming with ceramide-1 phosphate promotes the therapeutic effect of mesenchymal stem/stromal cells on pulmonary artery hypertension. *Biochemical and Biophysical Research Communications* 2016; **473:** 35-41.

4 Jin H, Bae Y, Kim M, Kwon S-J, Jeon H, Choi S *et al.* Comparative Analysis of Human Mesenchymal Stem Cells from Bone Marrow, Adipose Tissue, and Umbilical Cord Blood as Sources of Cell Therapy. *International Journal of Molecular Sciences* 2013; **14:** 17986.

5 Lee M, Jeong SY, Ha J, Kim M, Jin HJ, Kwon SJ *et al.* Low immunogenicity of allogeneic human umbilical cord blood-derived mesenchymal stem cells in vitro and in vivo. *Biochem Biophys Res Commun* 2014; **446:** 983-989.

6 Bibikova M, Lin Z, Zhou L, Chudin E, Garcia EW, Wu B *et al.* High-throughput DNA methylation profiling using universal bead arrays. *Genome research* 2006; **16:** 383-393.

7 Sandoval J, Heyn H, Moran S, Serra-Musach J, Pujana MA, Bibikova M *et al.* Validation of a DNA methylation microarray for 450,000 CpG sites in the human genome. *Epigenetics* 2011; **6:** 692-702.

8 Takai D, Jones PA. Comprehensive analysis of CpG islands in human chromosomes 21 and 22. *Proc Natl Acad Sci U S A* 2002; **99:** 3740-3745.

9 Irizarry RA, Ladd-Acosta C, Carvalho B, Wu H, Brandenburg SA, Jeddeloh JA *et al.* Comprehensive high-throughput arrays for relative methylation (CHARM). *Genome research* 2008; **18:** 780-790.

10 Bjornsson HT, Brown LJ, Fallin MD, Rongione MA, Bibikova M, Wickham E *et al.* Epigenetic specificity of loss of imprinting of the IGF2 gene in Wilms tumors. *Journal of the National Cancer Institute* 2007; **99:** 1270-1273.

11 Teschendorff AE, Marabita F, Lechner M, Bartlett T, Tegner J, Gomez-Cabrero D *et al.* A beta-mixture quantile normalization method for correcting probe design bias in Illumina Infinium 450 k DNA methylation data. *Bioinformatics (Oxford, England)* 2013; **29:** 189-196.

12 Mierzejewska K, Heo J, Kang JW, Kang H, Ratajczak J, Ratajczak MZ *et al.* Genome-wide analysis of murine bone marrow-derived very small embryonic-like stem cells reveals that mitogenic growth factor signaling pathways play a crucial role in the quiescence and ageing of these cells. *International Journal of Molecular Medicine* 2013; **32:** 281-290.

13 Shin DM, Zuba-Surma EK, Wu W, Ratajczak J, Wysoczynski M, Ratajczak MZ *et al.* Novel epigenetic mechanisms that control pluripotency and quiescence of adult bone marrow-derived Oct4+ very small embryonic-like stem cells. *Leukemia* 2009; **23:** 2042-2051.

14 Kucia M, Shin DM, Liu R, Ratajczak J, Bryndza E, Masternak MM *et al.* Reduced number of VSELs in the bone marrow of growth hormone transgenic mice indicates that chronically elevated Igf1 level accelerates age-dependent exhaustion of pluripotent stem cell pool: a novel view on aging. *Leukemia* 2011; **25:** 1370.

15 Kim A, Yu HY, Lim J, Ryu C-M, Kim YH, Heo J *et al.* Improved efficacy and in vivo cellular properties of human embryonic stem cell derivative in a preclinical model of bladder pain syndrome. *Scientific Reports* 2017; **7:** 8872.

16 Jez M, Ambady S, Kashpur O, Grella A, Malcuit C, Vilner L *et al.* Expression and Differentiation between OCT4A and Its Pseudogenes in Human ESCs and Differentiated Adult Somatic Cells. *PLOS ONE* 2014; **9:** e89546.

17 Jang YK, Kim M, Lee YH, Oh W, Yang YS, Choi SJ. Optimization of the therapeutic efficacy of human umbilical cord blood-mesenchymal stromal cells in an NSG mouse xenograft model of graft-versus-host disease. *Cytotherapy* 2014; **16:** 298-308.
